# The identification of new candidate genes Triticum aestivum *FLOWERING LOCUS T3‐B1* (*TaFT3‐B1*) and *TARGET OF EAT1* (*TaTOE1‐B1*) controlling the short‐day photoperiod response in bread wheat

**DOI:** 10.1111/pce.13018

**Published:** 2017-08-17

**Authors:** Meluleki Zikhali, Luzie U. Wingen, Michelle Leverington‐Waite, Sebastien Specel, Simon Griffiths

**Affiliations:** ^1^ John Innes Centre Norwich Research Park NR4 7UH Norwich UK; ^2^ Seed Co Limited, Rattray Arnold Research Station PO Box CH142 Harare Zimbabwe; ^3^ Limagrain Europe Centre de Recherche de Chappes Bâtiment 1, Route d'Ennezat 63720 Chappes France

**Keywords:** Brachypodium distachyon, GEDIFLUX, Watkins, Zea mays

## Abstract

Perception of photoperiod changes enables plants to flower under optimum conditions for survival. We used doubled haploid populations of crosses among Avalon × Cadenza, Charger × Badger and Spark × Rialto and identified short‐day flowering time response quantitative trait loci (QTL) on wheat chromosomes 1BS and 1BL. We used synteny between Brachypodium distachyon and wheat to identify potential candidates for both QTL. The 1BL QTL peak coincided with TaFT3‐B1, a homologue of the barley gene HvFT3, the most likely candidate gene. The 1BS QTL peak coincided with homologues of Arabidopsis thaliana S ENSITIVITY TO R ED LIGHT R EDUCED 1, WUSCHEL‐like and RAP2.7, which is also known as Zea mays TARGET OF EAT1, named TaSRR1‐B1, TaWUSCHELL‐B1 and TaTOE1‐B1, respectively. Gene expression assays suggest that TaTOE1‐B1 and TaFT3‐B1 are expressed more during short days. We identified four alleles of TaFT3‐B1 and three alleles of TaTOE1‐B1. We studied the effect of these alleles in the Watkins and GEDIFLUX diversity panels by using 936 and 431 accessions, respectively. Loss of TaFT3‐B1 by deletion was associated with late flowering. Increased TaFT3‐B1 copy number was associated with early flowering, suggesting that TaFT3‐B1 promotes flowering. Significant association was observed in the GEDIFLUX collection for TaTOE1‐B1, a putative flowering repressor.

## Abstract 2

We identify two novel short photoperiod wheat flowering time genes *TaFT3‐B1* and *TaTOE1‐B1* that promote and repress flowering, respectively. We show that *TaFT3‐B1*, the homologue of *Ppd‐H2* in barley, and *TaTOE‐B1* (less studies in the cereals) are likely candidates for the short‐day‐specific QTL that we report upon. The *TaTOE1‐B1* homologue in Arabidopsis thaliana is involved in the ageing pathway, suggesting that wheat and possibly other cereals use the same pathway. The four alleles of *TaFT3‐B1* and three alleles of *TaTOE1‐B1* we identified for these genes present alternative routes for the fine tuning of flowering time control in wheat.

## Introduction

The coinciding of flowering time with optimal conditions for seed set enhances the ability of plant species to survive and is also important to crop yield and global food security. The interaction between the genotype of a plant and its environment regulates response to seasonal changes. The genes that regulate perception of long photoperiods and their pathways are relatively well understood in long‐day temperate cereals. The major photoperiod response gene in the *Triticeae* is a pseudo response regulator first identified in barley (Turner *et al*. 2005), for which the three PHOTOPERIOD‐1 (*Ppd‐1*) homoeologues were discovered in wheat, *Ppd‐A1*, *Ppd‐B1* and *Ppd‐D1* (Beales *et al*. 2007; Wilhelm *et al*. 2009; Herndl *et al*. 2008). Dominant mutant alleles of these genes confer photoperiod insensitivity in wheat (day neutral) and are given a suffix *a* (*Ppd‐A1a*, *Ppd‐B1a* and *Ppd_D1a*, respectively); these cause early ear emergence under short days (SD). The recessive wild‐type alleles are given a suffix *b* and cause very late flowering unless exposed to long days (LD; Diaz *et al*. 2012; Beales *et al*. 2007; Wilhelm *et al*. 2009; McIntosh *et al*. 2003). UK wheat varieties are mostly photoperiod sensitive, with winter growth habit (vernalization requiring types).

Most studies of wheat flowering time networks were conducted in the diploid species Triticum monococcum and the tetraploid Triticum turgidum. The integrated wheat flowering model suggests that *Ppd‐1* is a promoter of flowering under LD by up‐regulating CO_2_, which, in turn, up‐regulates *VRN3* an orthologue of the Arabidopsis thaliana *FLOWERING LOCUS T1* (*FT1*) and rice *Hd3a* (Li *et al*. 2011; Higgins *et al*. 2010; Yan *et al*. 2006; Yan *et al*. 2003). Thus, *VRN3*/*FT1* is an integrator of the vernalization and photoperiodic pathways (Chen & Dubcovsky, 2012; Li *et al*. 2011; Dubcovsky *et al*. 2006). The *VRN2* gene has been shown to repress *VRN3/FT1* under LD (Li *et al*. 2011). Because transcript levels of *VRN2* are high during autumn when day length is still long, the integrated model postulates that *FT* is repressed by the high level of *VRN2* and prevents flowering during autumn (Distelfeld *et al*. 2009). The wheat flowering model proposed by Chen & Dubcovsky (2012) suggests that *CONSTANS* (*CO*) competes with *VRN2* for the *NUCLEAR FACTOR Y* (*NF‐Y*) subunit, which is needed by both genes to bind *FT*. In autumn, the high levels of *VRN2* relative to *CO* favour *VRN2* and *NF‐Y* binding, leading to floral repression, while the down‐regulation of *VRN2* by *VRN1* during vernalization in winter favours *CO* binding to *NF‐Y* complex resulting in floral induction in spring (Chen & Dubcovsky 2012).

However, less is currently understood about genes that regulate flowering during SD in wheat. The genes that regulate response to short photoperiod are mostly described for A. thaliana and rice, but little is known about how temperate cereals (wheat, barley, rye, oat and triticale) respond to SD (Milec *et al*. 2014; Shrestha *et al*. 2014; Chen & Dubcovsky 2012; Higgins *et al*. 2010). While *Ppd‐1* and *Ppd‐H1* account for some of the variation in flowering time under SD in wheat and barley, respectively (Turner *et al*. 2005; Beales *et al*. 2007; Wilhelm *et al*. 2009; Herndl *et al*. 2008), there is still genetic variation for photoperiod response in bread wheat that cannot be accounted for by *Ppd‐1* (Kumar *et al*. 2012). For example, Zikhali *et al*. (2014) reported that wheat cultivars Spark and Rialto are separated by about 2 week difference in flowering time under SD, although both carry the same photoperiod sensitive *Ppd‐1* alleles, suggesting that other loci could be responsible for short photoperiod response in wheat.

In barley, the photoperiod flowering response locus *Ppd‐H2* was shown to promote flowering under short‐day conditions and the proposed candidate gene was designated *HvFT3* (Faure *et al.* 2007). In the short‐day plant Zea mays, the *APETALA2‐like* gene *Glossy15* has been shown to be a repressor of flowering that acts by maintaining the juvenile phase (Lauter *et al*. 2005). Another *APETALA2‐like* gene called Z. mays *TARGET OF EAT1* (*ZmTOE1*) or *ZmRAP2.7*, a homologue of the *A. thaliana* gene *Related to APETALA2.7* (*RAP2.7*), plays a major role in Z. mays flowering time control (Dong *et al*. 2012; Higgins *et al*. 2010; Zhu & Helliwell 2011; Salvi *et al*. 2007 Okamuro *et al*. 1997).

Overexpression of *ZmTOE1* has been shown to delay flowering in maize as was observed for the related *AP2‐like* gene *Glossy15* (Salvi *et al*. 2007; Zhu & Helliwell 2011). To our knowledge, the homologues of *HvFT3* or *ZmTOE1* have not been cloned in wheat despite the crucial role that these two genes or their respective homologues play in flowering time in A. thaliana, Z. mays and barley (Hordeum vulgare).

We report here two quantitative trait loci (QTLs), one on 1BS (SD specific) and one on 1BL (observable under both short and LD), and propose as candidate genes *TaTOE‐B1* (a homologue of *ZmTOE1*) and *TaFT3‐B1* (a homologue of *HvFT3*), respectively. As *TaTOE‐B1* represses and *TaFT3‐B1* promotes flowering, these genes present alternative routes for the fine tuning of flowering time control in wheat.

## Materials and Methods

### Doubled haploid population growth conditions

Following the observation that Rialto was less sensitive to SD, flowering about 15 d earlier than Spark (Zikhali *et al*. 2014), this effect was investigated by using three doubled haploid (DH) populations of crosses among Spark × Rialto, Avalon × Cadenza and Charger × Badger. Ninety‐six lines each of the three independent DH populations were grown in 1 L pots. The growth conditions were as decribed by Zikhali *et al*. (2014). For each of the 96 DH lines from the three populations, nine seeds were sown and germinated between 15 and 20 °C for 2 weeks. The nine seeds of each line were then separated onto three photoperiod regimes (each had three plants of each line from the three DH populations), which were all initially set to 10 h of natural light. The plants in all treatments were vernalized for 8 weeks under SD (10 h light) at 6–10 °C by using natural vernalization in an unheated glasshouse.

After the 8 week vernalization period, one of the three photoperiod treatments remained unchanged and continued exposing plants to SD (10 h light). The others were adjusted to give LD (16 h light) and very LD (VLD, 20 h light). Augmentation of the 10 h of daylight provided in each treatment was achieved by additional 4 and 8 h artificial white light using 8 tungsten bulbs spaced 0.9 m apart delivering 1 mm s^−1^ m^−1^ to aid the LD and VLD, respectively. The temperature was maintained in the range of 13–18 °C. Days to ear emergence (DTEM) was measured on the leading tiller at Zadoks growth stage 55 (Zadoks *et al*. 1974). The DTEM scores were then used to carry out QTL analysis. QTL analysis on DTEM scores was conducted in r/qtl (vs. 3.02, R Core Team 2013) by using an equivalent of confidence interval mapping analysis.

### Comparative genomics exploiting synteny between Brachypodium and wheat

We used synteny between wheat and *Brachypodium* distachyon as well as the wheat (IWGSC‐based) pseudomolecule v3.3 (JIC) database to determine the gene order on group 1 chromosomes in areas spanning the QTL peak on 1BS and 1BL as described in an earlier report (Zikhali *et al*. 2015). We used blast homology searches of the wheat (IWGSC‐based) pseudomolecule v3.3 (JIC) by using sequences linked to markers on 1BS and 1BL QTL peaks and retrieved the positions of these markers on each of the pseudomolecules. We then used the pseudomolecule positions to align the QTL peak on 1BS for Avalon × Cadenza and the QTL peaks on 1BL for Charger × Badger, Spark × Rialto and Avalon × Cadenza with the syntenic genes in *B. distachyon* (*Brachypodium*), respectively. We used the same method to align the QTL identified by Kuchel *et al*. (2006) on 1A with the QTL on 1BL. The QTL by Kuchel *et al*. (2006) peaks between SSR markers *Xwmc304* and *Xgwm99*, which are also present in the Charger × Badger 1A map, which has additional KASP markers. We used the Charger × Badger KASP marker sequences to align the region spanning *Xwmc304* and *Xgwm99* with syntenic *Brachypodium* genes, which then aligned with the genes on 1BL.

### Assembly of the three wheat homologues and development

We assembled the three wheat homoeologues for *TaWUSCHELL‐B1*, *TaSRR1‐B1*, *TaTOE1‐B1* and *TaFT3‐B1* genes by using the method described by Zikhali *et al*. (2014). We then designed 1B‐specific primers (Table S2) to amplify these three genes as described by Zikhali *et al*. (2015). The primers were designed for *TaWUSCHELL‐B1*, *TaSRR1‐B1*, *TaTOE1‐B1* and *TaFT3‐B1*, having a 100% match with one of the sequences and to contain a maximum number of mismatches with the respective A and D homoeologues and ending with a 3′1B genome specific nucleotide. The 1B‐specific primers selectively amplified overlapping portions of *TaWUSCHELL‐B1*, *TaSRR1‐B1*, *TaTOE1‐B1* and *TaFT3‐B1* gene copies while competitively excluding A and D homoeologues.

### Amplification and sequencing of genes on 1B

Amplicons were obtained from genomic DNA for the *TaWUSCHELL‐B1*, *TaSRR1‐B1* and *TaFT3‐B1* genes by using the polymerase chain reaction (PCR) protocol and PCR reaction conditions and detected by agarose electrophoresis as described by Diaz *et al*. (2012) and Zikhali *et al*. (2014). The PCR was carried out in 20 *μ*L reactions comprising 2.5 *μ*L of 20 ng/*μ*L genomic DNA dissolved in 1× Tris‐EDTA (TE) buffer, 0.4 *μ*L of 10 mm deoxynucleotide (Promega UK LTD) dissolved in 1× TE buffer, 1.6 *μ*L of 25 mm MgCl2, 4.0 *μ*L of 5× clear buffer, 1 *μ*L each of 5 *μ* m (dissolved in 1× TE buffer) forward and reverse primers, 0.08 *μ*L GO TAQ FLEXI DNA (Promega UK LTD) polymerase (5 U/*μ*L) and 9.42 *μ*L of double distilled water.

We modified this standard PCR protocol to sequence the GC rich region in the first exon of *TaTOE1‐B1*, which could not be amplified by using the standard PCR protocol. The modification used 1.5 *μ*L of either ethylene glycol or 1,2‐propanediol, both solvents shown to aid amplification of GC‐rich human genomic DNA (Zhang *et al*. 2009). We then reduced the amount of water from 9.42 to 7.92 *μ*L to maintain a reaction volume of 20 *μ*L. The amplicons were directly sequenced for Spark, Rialto, Avalon, Cadenza, Charger, Badger, Malacca, Hereward, Claire and Savannah by using ABI Big Dye Mix v3.1 (Applied Biosystems Inc.) under the manufacturer's conditions, with products resolved on an ABI 3730 capillary electrophoresis instrument.

### KASP genotyping

DNA extractions and KASP SNP genotyping were essentially carried out as in Knight *et al*. (2015) by using specific primers for *TaFT3‐B1* and *TaTOE1‐B1* (Table S3) designed in this study. All KASP™ amplifications were carried out in 1536‐well plates by using 1 *μ*L of KASP™ master mix 1X (LGC group, UK) and 0.0135 *μ*L of primer mix (12 *μ*L FAM primer at 100 *μ* m + 12 *μ*L of VIC primer at 100 *μ* m + 30 *μ*L of common primer at 100 *μ* m + 46 *μ*L of dH_2_O). One microlitre of DNA at 2 ng/*μ*L was previously added on each well of the 1536 plates and dried at 60 °C for 30 min. PCR reactions were carried out by using a touchdown program: 95 °C for 15 min, then 10 cycles of 95 °C for 20 s and 61 °C for 60 s (−0.6 °C per cycle), followed by 26 cycles of 95 °C for 20 s and 55 °C for 60 s on a hydrocycler.

### Copy number variation determination

Copy numbers of *TaFT3‐B1* were detected following the protocol described by Díaz *et al*. (2012), using labelled probes for *TaFT3‐B1*. The primers and probes were designed by using applied primer express software with primers that were genome and locus specific and common TaqMan^®^ MGB probes for each target. To quantify copy number variation on *TaFT3‐B1*, PCRs were made in Duplex by using *TamyB* gene as internal endogenous control. PCR reaction was carried out in 384‐well PCR plates by using 4 *μ*L of 2 ng/*μ*L genomic DNA dissolved in purified water, 5 *μ*L of KlearKall (LGC group, UK) Master mix (2X), 0.09 *μ*L each of 100 *μ* m forward and reverse primers (endogenous + target), 0.0125 *μ*L of 100 *μ* m VIC‐labelled probe for endogenous, 0.0125 *μ*L of 100 *μ* m FAM‐labelled probe for Target and 0.615 *μ*L of double distilled water for a total of 10 *μ*L PCR reaction. The amplification and fluorescence collection were made on the 7900HT Real‐Time PCR System by using program: 95 °C for 10 min, then 40 cycles of 95 °C for 15 s, 61 °C for 25 s and 72 °C for 25 s. The result analysis and calculation of delta CT values were made by using applied rq manager ^®^ software.

### Gene expression

Four sets of plants containing three individuals each for Spark, Rialto, Avalon, Cadenza, Charger, Badger, Savannah and Chinese Spring were grown under SD (10 h light) at 5–8 °C (vernalization treatment). All above ground parts of 3‐week‐old plants from the first set were harvested and ground by using a pestle and mortar. Three plants were combined for each sample. Samples were collected at 10.00 am during the light period. A second set was harvested similarly after 4 weeks. After 8 weeks of vernalization, the two other sets were moved into different controlled environments, one under SD (10 h light and 14 h darkness) and the other under LD (16 h light and 8 h darkness), and both environments were kept at 16–18 °C in the light and 13–15 °C in the dark period. Five weeks after vernalization (week 13), both plant sets were processed as described in the preceding texts. Expression studies and analysis of *TaTOE1‐A1*, *TaTOE1*‐*B1* and *TaTOE1‐D1*; *TaFT1‐A1*, *TaFT1‐B1* and *TaFT1‐D1*; and *TaFT3‐A1*, *TaFT3‐B1* and *TaFT3‐D1* were carried out as described for *TaGI* and *TaELF3* by Zikhali *et al.* (2015) by using *norm2* forward primer agcgatttccagctgccttc and reverse primer tgcgaagaggccagtcagtc as the reference gene. Optimal genome‐specific qPCR assays were developed for *TaFT3‐A1*, *TaFT3‐B1* and *TaFT3‐D1* as well as for *TaTOE1‐A1*, *TaTOE1‐B1* and *TaTOE1‐D1*. For *TaFT1‐A*, *TaFT1‐B* and *TaFT1‐D*, we used primers developed by Shaw *et al*. (2012).

### Watkins and GEDIFLUX diversity panels

We used DTEM scores for the years 2006 and 2011 reported by Wingen *et al.* (2014) from the Watkins and GEDIFLUX diversity panels with 936 and 418 accessions, respectively, to study the effect of the alleles of *TaFT3‐B1* and *TaTOE1‐B1* on flowering time. In addition to that, we also used DTEM data for the Watkins and Gediflux collections from 2014 and 2016 (Morley), respectively. Both trials were non‐replicated trials in 6 m^2^ plots. The Watkins panel is a landrace collection from 32 countries compiled in the 1930s. The Gediflux collection consists of elite European wheat germplasm selected on the basis that each entry occupied at least 5% of the winter wheat acreage in a North European country in the period 1945–2000.

### Statistical analysis

A general linear model analysis in tassel software (Bradbury *et al*. 2007), version 5, was conducted following the methods in N'Diaye *et al.* 2017, to control for spurious associations, population structure and/or relatedness between individuals. The general linear model analysis was conducted on Axiom genotype data for chromosome 1B available from CerealsDB (http://www.cerealsdb.uk.net) together with the genotype scores for *TaTOE1‐B1 and TaFT3‐B1*. The Q matrices were based on discriminant analysis of principal components on the SSR data from Wingen *et al*. (2014). These matrices were based on nine groups for the Watkins collection, to reflect the ancestral groups, and on six groups for the Gediflux collection. For both collections, the respective kinship matrix was calculated by using tassel and the SSR data. A significant outcome was defined by a false discovery rate < 0.01.

## Results

### The Avalon × Cadenza 1BS short‐day‐specific QTL

**Figure 1:**
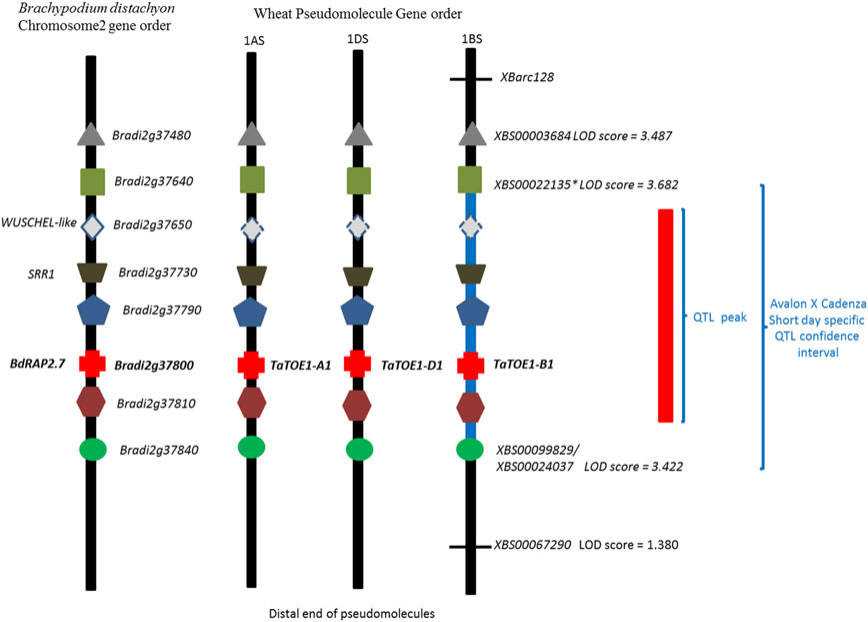
Schematic presentation of the Avalon × Cadenza short‐day‐specific flowering quantitative trait locus (QTL) on chromosome 1BS showing conserved gene order with homoeologous regions on1DS and 1As and Brachypodium distachyon chromosome 2. The vertical black rectangles represent chromosomes, and the coloured shapes on the black rectangles represent the equivalent regions of *Brachypodium distachyon* and chromosomes 1AS, 1BS and 1DS. The blue rectangle on 1BS represents QTL confidence interval defined by markers XBS00022135 and XBS00099829, and the red vertical rectangle denotes the peak (logarithm of the odds score above 3.9) of the QTL between markers XBS00022135 and XBS00099829. The marker that accounts for most of the variation is marked with an asterisk. *WUSCHEL‐like* denotes the B. distachyon putative *WUSCHEL‐related homeobox 2* gene (*Bradi2g37650*). The dashed line for the WUSHEL‐like gene for 1AS, 1DS and 1Bs pseudomolecules is used because this gene was not assigned to the draft assembly, but they are all located on the short arm of group 1 chromosomes. SSR1 denotes the predicted B. distachyon *SENSITIVITY TO RED LIGHT REDUCED 1* gene (Bradi2g37730). BdRAP2.7 denotes the B. distachyon RELATED to APETALLA 2.7, also known as *TARGET OF EAT* (*EARLY ACTIVATED TAGGED*) *1* (*TOE1*; Higgins *et al*. 2010).

We identified an SD‐specific QTL on chromosome 1BS in the Avalon × Cadenza DH population (Fig. 1). The peak of this QTL was between KASP markers *XBS00022135* and *XBS00099829* (Allen *et al*. 2011) that match the *Brachypodium* chromosome 2 genes *Bradi2g37640* and *Bradi2g37840*, respectively (Fig. 1). There are 19 genes between *Bradi2g37640* and *Bradi2g37840* in *Brachypodium*, and 15 of these 19 genes have sequence matches with wheat group1 short arm (1AS, 1BS and 1DS) genes (Table S1). Based on the function of these 15 genes in *Brachypodium* and other species, only three of these – *TaBradi2g37650*, a putative *WUSCHEL‐related homeobox 2* gene (Laux *et al*. 1996); *TaBradi2g37730*, a predicted *Brachypodium* homologue of A. thaliana *SENSITIVITY TO RED LIGHT REDUCED 1* (Staiger *et al*. 2003); and *TaBradi2g37800*, an *APETALA2.7*‐like gene (Higgins *et al*. 2010; Okamuro *et al*. 1997) – have been reported to affect flowering time (Fig. 1 & Table S1). We named the wheat equivalent of these three *Brachypodium* genes on the chromosome 1B homologue as Triticum aestivum *WUSCHEL‐like* (*TaWUSCHELL‐B1*), T. aestivum *SENSITIVITY TO* R*ED LIGHT REDUCED 1* (*TaSRR1‐B1*) and T. aestivum *TARGET OF EAT1* (*TaTOE1‐B1*), respectively. Because the three genes are in the region of the QTL peak (Fig. 1), we prioritized these as potential candidates and sequenced them.

### Sequencing *TaWUSCHELL‐B1* and *TaSRR1‐B1*

There were no differences between the Avalon and Cadenza *TaWUSCHELL‐B1* and *TaSRR1‐B1* gene sequences in the open reading frame or the 139bp of sequence upstream of the start codon. We do not rule out the possibility of mutations within the promoter upstream of the 139 bases that we sequenced.

### Sequencing *TaTOE1‐B1*

**Figure 2:**
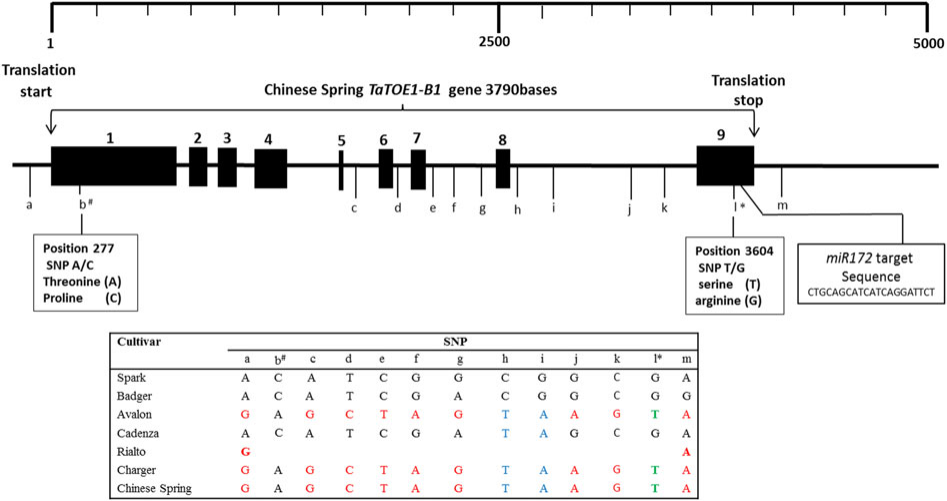
Schematic presentation of the *TaTOE1‐B1* gene. The black rectangles numbered 1–9 are the exons, and the introns are the spaces between the numbered rectangles. The lower case letters a–l denote the position of the single nucleotide polymorphisms (SNPs) in the gene sequences of cultivars Spark, Badger, Avalon, Cadenza, Rialto, Charger and Chinese Spring. The uppercase letters A, T, C and G represent DNA bases adenine, thymine, guanine and cytosine, respectively. The coloured letters denote the haplotype associated with early flowering. b# and l* denote the SNPs likely to affect function because SNP A–C change the amino acid threonine to proline and the SNP G‐T changes the wild‐type amino acid serine to arginine. The position of the *TamiR172* target sequence in exon 9 is shown. [Colour figure can be viewed at wileyonlinelibrary.com]

Sequencing *TaTOE1‐B1* for Avalon and Cadenza revealed 10 SNPs between the gene sequences from these two cultivars (Fig. 2). One of these SNPs was −440 bases upstream of the start codon (a), seven of the SNPs were in the introns, two were in the exons (Fig. 2b^#^,l*), and the last was 289 bases downstream of the stop codon (Fig. 2). The mutation in the first exon changes threonine to proline, while the mutation in the last exon changes serine to arginine (Fig. 2). We genotyped‐by‐sequencing the gene in other cultivars, including Claire, Malacca, Hereward and Savannah that showed delayed flowering under SD. The SNPs separate the early flowering Rialto and Charger cultivars from the late flowering Spark, Claire, Malacca, Badger, Hereward and Savannah wild‐type winter wheat cultivars (Table 1). Cultivar Rialto seemed not to have larger parts of the gene sequence because only amplicons from the promoter and part of the last exon were amplified, suggesting a deletion within this gene (Fig. 2).

**Table 1:** The genotype of nine winter wheat cultivars and one spring wheat cultivar (Cadenza) at five genes affecting flowering time. The cultivars were fully vernalized (8 weeks at 5–8 °C) and then grown in a controlled environment giving a daily cycle of 10 h light and 14 h darkness. The numbers in the SD Hd (short‐day heading date) row are the days after 1 May for the cultivars to reach Zadoks growth stage 55 (Zadoks *et al*. 1974) that is 50% ear emergence out of the flag leaf on the leading tiller

Gene	Spark	Claire	Malacca	Badger	Hereward	Savannah	Avalon	aCadenza	Rialto	Charger
*TaFT3‐B1*	mut	mut	WT	WT	mut	mut	mut	WT	WT	mut
*TaTOE1‐B1*	WT	WT	WT	WT	WT	WT	mut	WT	mut	mut
*TaELF3‐D1*	mut	–	–	mut	–	mut	WT	mut	WT	WT
*TaELF3‐B1*	WT	–	–	WT	–	WT	mut	WT	WT	WT
*Vrn‐A1* CNV	1	1	2	2	3	2	2	1	2	2
SD Hd	69	66	65	65	65	63	59	53	50	49
		Late flowering					Middle	Early flowering		

WT, wild‐type functional gene; mut, loss of function mutation or mutation likely to affect function in the open reading frame; SD Hd, short‐day heading date (10 h light and 14 h darkness); CNV, copy number variation; –, genotype not determined.

a Spring wheat.

### 1BL photoperiod QTL

**Figure 3:**
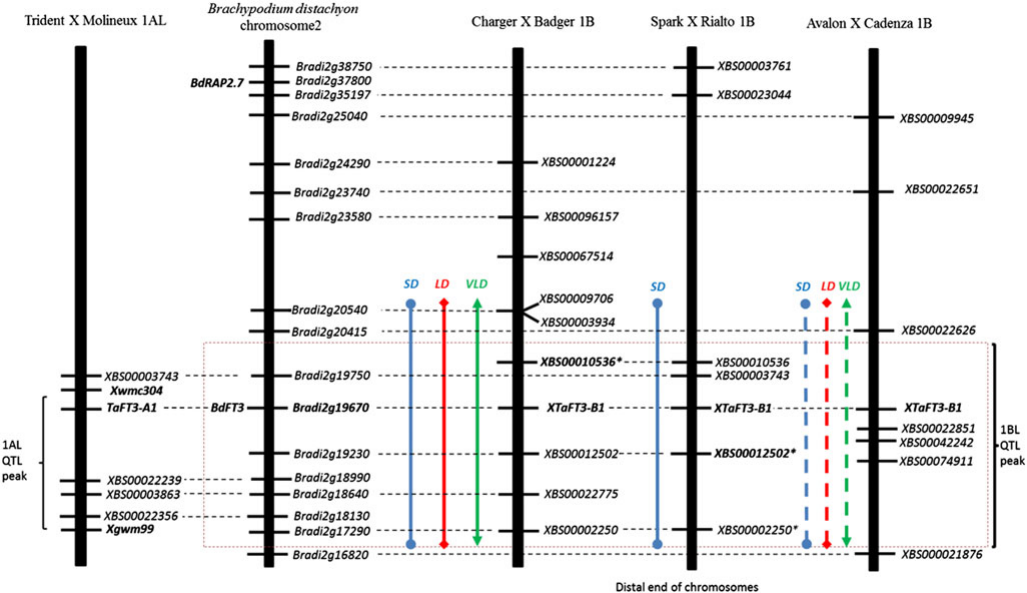
Schematic presentation of the Charger × Badger, Spark × Rialto and Avalon × Cadenza doubled haploid (DH) populations flowering quantitative trait locus (QTL) on 1BL. The Trident × Molineux QTL interval on 1AL defined by markers Xwmc304 and Xgwm99 was identified by Kuchel *et al*. (2006) and aligns to the same locus as the QTLs on 1BL, suggesting that these QTLs are likely homologous. The QTL was significant under short days (SD, 10/14 h light), long days (LD, 16 h light) and very long days (VLD, 20 h light) for the Charger × Badger population denoted by the blue, red and green vertical solid lines, respectively. The QTL was SD specific for the Spark × Rialto population. The Avalon × Cadenza population behaved like Charger × Badger, except that all the QTLs were below the significance threshold denoted by the dashed blue, red and green vertical lines. The dotted horizontal lines link KASP markers that have sequence matches with the syntenic Brachypodium distachyon chromosome 2 genes. The solid vertical bars represent the B. distachyon chromosome two gene order and marker order for the chromosome 1B Charger × Badger, Spark × Rialto and Avalon × Cadenza maps, respectively. The asterisk accounts for most of the variation. The QTL images for all the three populations are shown in Fig. S1. [Colour figure can be viewed at wileyonlinelibrary.com]

We also identified a QTL on 1BL that was present in all the three photoperiod treatments (SD, LD and VLD) in the Charger × Badger DH population. At the equivalent location, a QTL in the Spark × Rialto DH population (Fig. 3) was SD specific. An additional QTL at this location in the Avalon × Cadenza population behaved like the Charger × Badger QTL but was below the significance threshold in the three photoperiod regimes (Fig. 3). Using synteny between wheat and *Brachypodium*, we identified the gene *TaFT3‐B1*, a homologue of the barley gene *HvFT3*, as a possible candidate for these effects because this gene has been shown to affect flowering time particularly under SD in barley (Faure *et al*. 2007). Furthermore, we mapped the gene between KASP markers *XBS00010536* and *XBS00012502*, which account for most of the variation in the Charger × Badger and Spark × Rialto DH populations, respectively (Fig. 3). The gene is located in the peak region of the QTL in all the three DH populations (Figs S1 & S2). Moreover, the 1BL QTL locus and a QTL identified on 1AL by using spring wheat (Fig. 3; Kuchel *et al*. 2006) seem to be in syntenic regions, suggesting that homologues genes may be responsible for the two flowering time QTL.

### Mutations in the *TaFT3‐B1* gene

**Figure 4:**
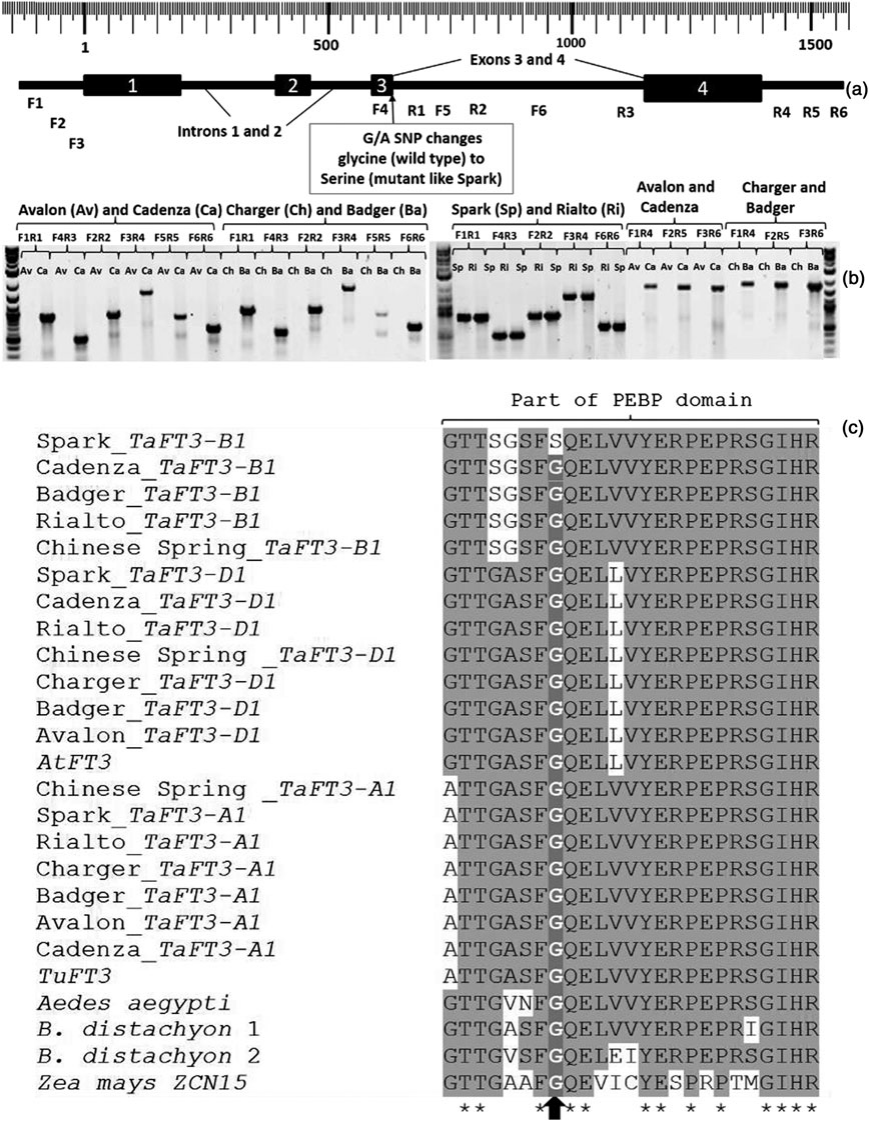
Schematic representation of the *TaFT3‐B1* gene (a), polymerase chain reaction (PCR) amplicons of *TaFT3‐B1* (b) and the conserved amino acid glycine that is mutated to serine in Spark (c). The position of the B genome‐specific PCR primers (F1‐R6) along the gene (a) and the position of the single nucleotide polymorphism (SNP) at the last base of exon 3 (b) that changes a conserved glycine (wild type) to serine (mutant Spark) are shown. Agarose gel electrophoresis separation of *TaFT3‐B1* PCR amplicons (b) from Avalon (Av) Cadenza (Ca), Charger (Ch), Badger (Ba) Spark (Sp) and Rialto (Ri). The gene is deleted in Avalon (Av) and Charger (Ch) but is intact in Cadenza (Ca), Spark (Sp) and Rialto (Ri). The Spark point mutation G/A changes (c) a highly conserved amino acid glycine (G) to serine (S) in the PEBP domain of the *TaFT3‐B1* gene shown by the black upward facing arrow. *TaFT3* = Triticum aestivum *FT3*, CS = Chinese Spring, *AtFT3* = Aegilops tauschii, *TuFT3* = Triticum urartu *FT3*, B. distachyon 1 and 2 = Brachypodium distachyon *HEADING DATE 3A* and *3B‐like* GenBank accession numbers XM_003569759 and XM_003568040, respectively. The *ZCN1*5 = Zea mays *CENTRORADIALIS15* Genbank accession EU241906.

Agarose gel electrophoresis of PCR amplicons obtained by using *TaFT3‐B1* gene‐specific primers (Table S1) as well as sequencing of the PCR amplicons revealed that Avalon and Charger have lost the *TaFT3‐B1* gene (Fig. 4b). Four variations of the *TaFT3‐B1* gene were identified. The first allele is the wild‐type functional gene sequence detected in cultivars Cadenza, Badger and Rialto (Fig. 4c) and is associated with early flowering (Figs 6 & S3). The second allele is a deletion of the whole *TaFT3‐B1* gene in Avalon and Charger (Fig. 4b) and is associated with the late flowering phenotype (Figs 6 & S3). The third allele, which is also associated with the late flowering phenotype (Fig. S3), is the SNP that causes an amino acid change (glycine–serine) in the Spark sequence (Fig. 4a,b). The glycine (wild type) is conserved across all the three homoeologues and homologues from different species including Z. mays, Sorghum bicolor, B. distachyon and *Aedes aegypti* (Fig. 4c)*.* This glycine, which is in the ligand‐binding motif of the phosphatidylethanolamine‐binding protein domain (Danilevskaya *et al*. 2008), was also shown to be conserved in 5 barley *FT* genes (*HvFT1*, *HvFT2*, *HvFT3*, *HvFT4* and *HvFT5*), 14 Oryza sativa *FT* genes and the A. thaliana *FT* gene (Faure *et al*. 2007). Sequence alignment of the 25 PEBP genes known as Z. mays *CENTRORADIALIS* (*ZCN1–26*), numbered 1 to 26 because there is no *ZCN22*, reveal that all except *ZCN25* have the conserved glycine (Fig. 3b; Danilevskaya *et al*. 2008) that is mutated in Spark, suggesting that the glycine‐to‐serine mutation in Spark is likely to affect function. In addition to that, the *ZCN25* gene has low transcript levels, while its close paralogue *ZCN19* is highly expressed (Danilevskaya *et al*. 2008), suggesting that this gene, which has a mutation at the same conserved amino acid as Spark, may have lost part or all of its function. Additional to these three different alleles of *TaFT3‐B1*, we also found copy number variations of two versus six copies of this gene.

**Figure 5:**
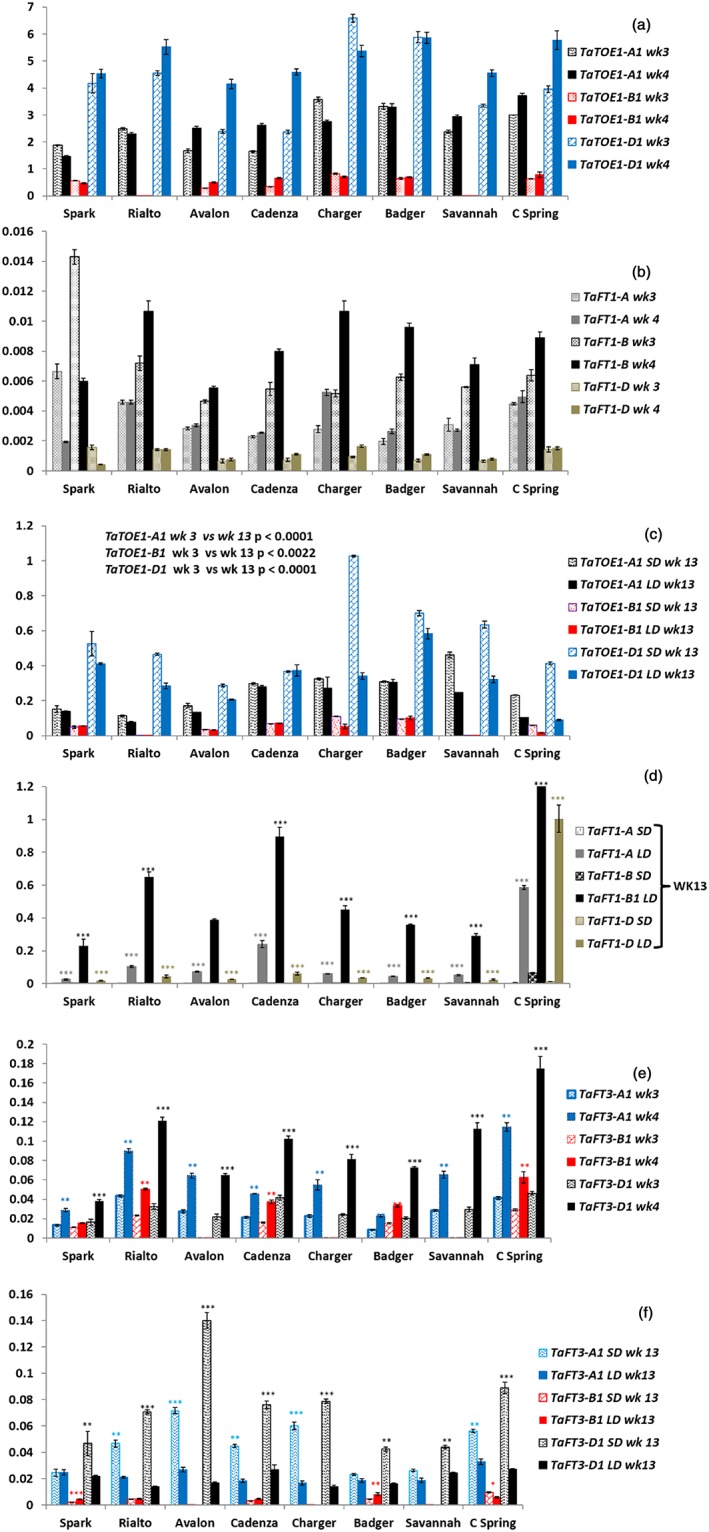
Gene expression patterns of the *TaTOE1* (a and c) and *TaFT1* (b and d) and *TaFT3* (e and f) homoeologues relative to *NORM2* expression at 3, 4 and 13 weeks (wk) after planting. The experiments at weeks 3 and 4 were done under short days (10 h light and 14 h darkness), while the experiments at week 13 were done under both short and long days designated SD and LD, respectively. *P < 0.01, **P < 0.001, ***P < 0.0001. The error bars are the standard error of the mean. For Fig. 5d, the significant differences are the differences in means under short days relative to long days for the *TaFT1* homoeologues; for Fig. 5e, the significant differences are the differences in mean expression of the *TaFT3* homoeologues between weeks 3 and 4, while for Fig. 5f, the significant differences were measured for the mean expression of *TaFT3* homoeologues under short relative to long days. We also compared the expression patterns of the *TaTOE1* homologues at week 3 relative to week 13, and the following *P*‐values were obtained: *TaTOE1‐A1* (*P* < 0.0001), *TaTOE1‐B1* (*P* < 0.0022) and *TaTOE1‐D1* (*P* < 0.0001), and expression was higher at week 3 relative to week 13 for all the three homoeologues with approximately a 10‐fold reduction in expression at week 13 relative to week 3 for the three homoeologues (a and c). For *TaFT1*, expression was approximately 50‐fold higher under long days at week 13 relative to the average of weeks 3 and 4 with *P* < 0.0001 (b and d). [Colour figure can be viewed at wileyonlinelibrary.com]

Because the Spark × Rialto 1BL QTL was SD specific while the QTL at the same locus for Charger × Badger was observed in SD as well as LD (Fig. 3), one hypothesis was that Badger may have a mutation in the promoter that causes the Badger *TaFT3‐B1* gene to be differently regulated, resulting in expression even in LD. This possibility was checked by sequencing the *TaFT3‐B1* gene 1380 bases upstream of the start codon, but no polymorphism between the Badger allele and alleles of other cultivars was found. However, it is interesting to note that, in Badger, the expression of *TaFT3‐B1* is twofold higher than in the other cultivars (*P* < 0.0001) under LD (Fig. 5f), which could be the reason why the Charger × Badger DH population has the QTL under both SD and LD.

### Comparison of the effect of *TaFT3‐B1* and *TaTOE1‐B1* mutations

Having identified two genes, *TaTOE1*‐*B1* (a putative flowering repressor) and *TaFT3‐B1* (a putative flowering promoter), that are likely to affect flowering time under SD, and for *TaFT3‐B1* possibly also under LD, we compared the phenotypes of cultivars that had different combinations of these genes (Table 1). Charger flowers earlier than Badger; however, it can be suggested that the Charger *TaFT3‐B1* allele contributes towards lateness (Fig. S1a); hence, the earliness of Charger may be attributed to the mutant *TaTOE1‐B1* allele (Figs 2 & S1 & Table 1) or other genes in the genetic background. Cadenza flowers early under SD even though it has the floral repressor *TaTOE1‐B1* wild‐type allele, possibly because of its spring background. Flowering time of Avalon lies between the early and late flowering cultivars and, interestingly, it has lost both the floral promoter *TaFT3‐B1* and the floral repressor *TaTOE1‐B1* (Table 1).

### Diversity panels Watkins and GEDIFLUX

**Figure 6:**
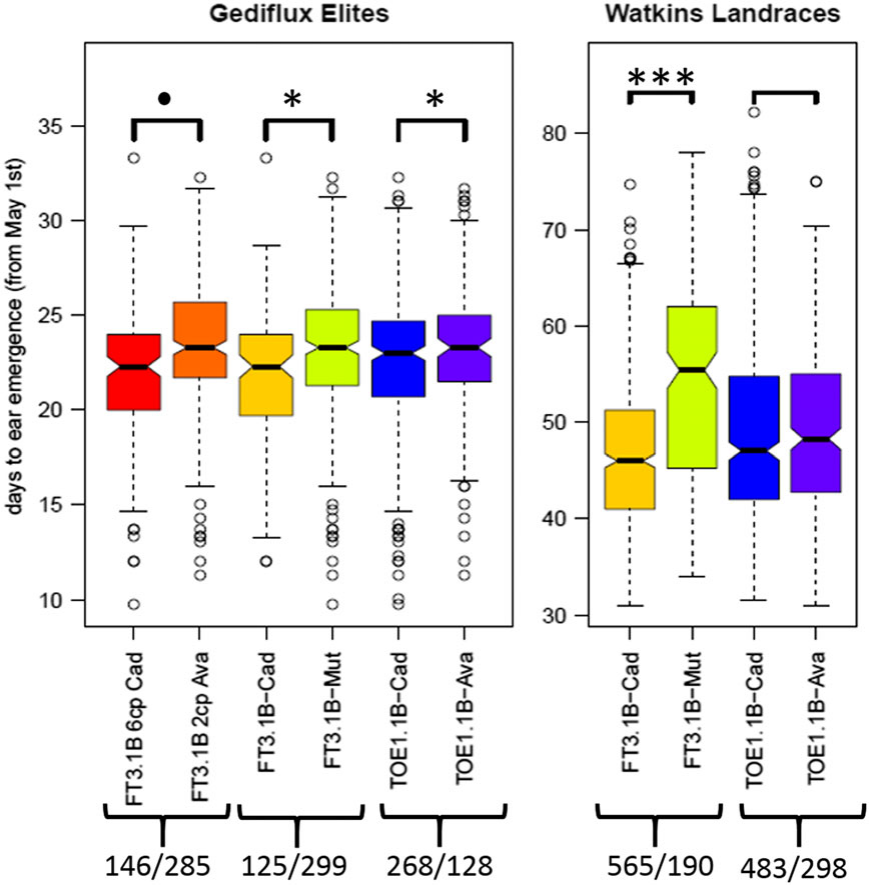
Box plot of the distributions of days to ear emergence (DTEM) for the GEDIFLUX elite collection of European winter wheat and Watkins landrace collection carrying different alleles of *TaFT3‐B1* and *TaTOE1‐B1* genes. The allele ratios described in the legend are shown with a forward slash (/). One hundred forty‐six accessions in the GEDIFLUX had six copies of Cadenza‐type *TaFT3‐B1* (FT3.1B‐6cp‐Cad), while 285 accessions had two copies *TaFT3‐B1* copies of the Avalon type (FT3.1B‐2cp‐Ava). Two hundred sixty‐eight accessions in the GEDIFLUX collection had the Cadenza‐type allele of *TaTOE1‐B1* (TOE1.1B‐Cad), while 128 accessions had the Avalon *TaTOE1‐B1* allele type (TOE1.1B‐Ava). For the Watkins collection, 565 accessions had the Cadenza‐type *TaFT3‐B1* (FT3.1B‐Cad) allele, while 190 had the Avalon/Spark allele. For *TaTOE1‐B1*, the Watkins collection had 483 and 298 Cadenza and Avalon allele types, respectively. DTEM data collection is described in Wingen *et al.* (2014). Statistically significant differences in DTEM, detected in a GLM analysis, were found between accessions carrying different alleles of *TaFT3‐B1* in both collections and of *TaTOE1‐B1* in the Gediflux collection. Significance levels are given above the vertical square brackets at the top of the plot: no star = p.adj > 0.05, **. =** p.adj < 0.05, * = p.adj < 0.01, *** = p.adj < 0.001. [Colour figure can be viewed at wileyonlinelibrary.com]

In the Watkins panel, the *TaFT3‐B1* allele was significantly associated with flowering time (*P* < 0.0001; Fig. 6). The loss‐of‐function *TaFT3‐B1* alleles, either a deletion or an SNP that changes the coding for a strongly conserved amino acid (glycine to serine), were both associated with late flowering, while the wild type was early flowering (Figs 4 & 6). Increased copy number of *TaFT3‐B*1 was also associated with early flowering (Fig. 6). For the GEDIFLUX panel, again, the *TaFT3‐B1* was significantly associated with flowering time (*P* < 0.0001) in the same manner as the Watkins collection (Fig. 6). The flowering data for the Watkins collection are in Table S4.

For *TaTOE1‐B1* gene, the Avalon allele was associated with late flowering, while the Cadenza polymorphism was associated with early flowering (*P* = 0.0181) in the GEDIFLUX collection. There was no significant difference in the Watkins collection for the *TaTOE1‐B1* allele. Given that the landrace collection is more genetically diverse than the elite germplasm, we suggest that the lack of significance in the Watkins collection maybe due to other background genes masking the *TaTOE1‐B1* effect.

### *TaTOE1*, *TaFT1* and *TaFT3* expression analysis

For *TaTOE1*, *TaTOE1‐D1* was the most expressed followed by *TaTOE1‐A1* (Fig. 5a,c). The *TaTOE1* homoeologues were expressed approximately 10‐fold higher in the juvenile phase (Fig. 5a) relative to the adult phase (Fig. 5c). The expression of *TaTOE1*, though reduced in the adult phase, was also higher during the SD relative to the LD (Fig. 5c).

For *TaFT1*, *TaFT1‐B* was the most expressed followed by *TaFT1‐A* (Fig. 5b,d). The expression of *TaFT1* was 100‐fold lower in the juvenile phase relative to the adult phase (Fig. 5b,d). The 100‐fold increase in *TaFT1* expression coincides with a 10‐fold reduction in *TaTOE1* expression in the adult phase relative to the juvenile phase (Fig. 5a–c). In the adult phase, *TaFT1* expression was low under SD, but significant (*P* < 0.0001) expression was detected in LD for all three homoeologues across all the wheat cultivars (Fig. 5d). PCR amplification (data not shown) suggests that Rialto has a deletion in the *TaTOE1‐B1* coding region as no amplification was achieved (Fig. 5a,c). The gene expression data show that this gene is not expressed in Savannah or Rialto (Fig. 5a,c), suggesting that Rialto and Savannah lack *TaTOE1‐B1*.

For *TaFT3*, *TaFT3‐D1* was the most expressed followed by *TaFT3‐A1* (Fig. 5e,f). PCR amplification suggested that Avalon, Charger and Savannah do not have a copy of *TaFT3‐B1* (Fig. 4b), and the lack of expression (Fig. 5e,f) supports this. The expression of all the *TaFT3* homoeologues was significantly increased at week 4 relative to week 3, except for Spark *TaFT3‐B1* (Fig. 5e). The expression of *TaFT3‐D1* and *TaFT3‐A1* was significantly higher under SD relative to LD, except for Spark and Badger *TaFT3‐A1* (Fig. 5f). For the parents of the DH populations where the 1BL flowering QTL was initially detected, Badger had twice the expression level of *TaFT3‐B1* under LD relative to Spark, Rialto and Cadenza (Fig. 5f). Spark and Rialto had no significant difference in the expression of *TaFT3‐B1* under LD, and expression is generally lower than A and D (Fig. 5f).

## Discussion

Our results suggest that *TaTOE1‐B1* is a more likely candidate than *TaSRR1‐B1* and *TaWUSCHELL‐B1* for the 1BS SD‐specific flowering time QTL and that *TaFT3‐B1* is a likely candidate for the 1BL flowering time QTL. These two genes have contrasting effects where *TaTOE1‐B1* is a floral repressor under SD and *TaFT3‐B1* is a floral promoter under SD and LD depending on the genetic background (Figs 1 & 3).

## The 1BS QTL Candidates

### Possibility that *TaWUSCHELL‐B1* is the candidate for 1BS QTL

The gene *TaBradi2g37650* is a putative *WUSCHEL‐related homeobox 2* that is required for proper shoot and floral meristem development (Laux *et al*. 1996). The lack of polymorphism between Avalon and Cadenza in the open reading frame and promoter of *TaWUSCHELL‐B1* coupled with the absence of any report in literature linking mutants at this gene with flowering time variation led us to conclude that *TaWUSCHELL‐B1* was not a candidate for the SD‐specific QTL.

### Possibility that *TaSRR1‐B1* is the candidate for 1BS QTL

The A. thaliana *SRR1* gene is crucial for normal circadian clock function by targeting clock genes *CCA1* and *TOC1* (Staiger *et al*. 2003). The A. thaliana *ssr1* mutants flower very late under SD relative to the wild type, although the difference is not significant under LD (Staiger *et al*. 2003). Even though mutation at this gene in A. thaliana accelerates flowering under SD (Staiger *et al*. 2003), the identity of the Avalon and Cadenza open reading frame sequences of *TaSRR1‐B1* led us to conclude that *TaSRR1‐B1* is an unlikely candidate for the Avalon × Cadenza SD‐specific QTL (Figs 1 & S1), although we do not rule out the possibility of promoter mutations upstream of the 139 bases from the translation start codon we sequenced.

### *TaTOE1‐B1* is the more likely candidate for 1BS short‐day‐specific flowering time QTL

The gene *TaBradi2g37800* is a homologue of the A. thaliana gene *RAP2.7* and Z. mays *ZmTOE1* or *ZmRAP2.7* (Dong *et al*. 2012; Higgins *et al*. 2010; Zhu & Helliwell 2011; Salvi *et al*. 2007; Okamuro *et al*. 1997). The polymorphism between Avalon and Cadenza at the *TaTOE1‐B1* gene (Fig. 2), as well as the separation of early and late flowering cultivars by using the SNPs in this gene (Table 1), together with the documented role of homologues of this gene in regulating flowering in A. thaliana and the SD plant Z. mays (Dong *et al*. 2012; Higgins *et al*. 2010; Zhu & Helliwell 2011; Jung *et al*. 2011; Salvi *et al*. 2007; Okamuro *et al*. 1997), led us to suggest that *TaTOE1‐B1* was the likely candidate for the SD‐specific QTL.

In A. thaliana, *RAP2.7*, the homologue of *TaTOE1‐B1*, is involved in the ageing pathway where it acts as a repressor of *FT* (Jung *et al*. 2007). *RAP2.7* is repressed by *miR172*, which binds to its mRNA, hence preventing translation in the adult phase (Zhu and Halliwell, 2011; Higgins *et al*. 2010; Aukerman & Sakai, 2003). Our results suggest that *TaTOE1‐B1* is a repressor of flowering, given that the mutants of this gene are early flowering (Fig. 2 & Table 1), an observation which is consistent with studies done in both A. thaliana and Z. mays. Our work is another step towards understanding the gene network that regulates flowering under SD in wheat. It is tempting to speculate at this point that wheat may have a similar pathway for this gene as A. thaliana or maize, but this hypothesis needs to be tested by developing near isogenic lines for the 1BS QTL and performing gene expression assays for the wheat homologues of the A. thaliana genes involved in the ageing pathway.

## The 1BL QTL Candidate

### *TaFT3‐B1* as possible candidate for 1BL QTL

The results for Spark × Rialto *TaFT3‐B1* QTL are consistent with studies from barley, a plant with a very similar photoperiod pathway to wheat, which showed that *HvFT3* is expressed mostly under SD (Laurie *et al*. 1995; Faure *et al*. 2007). In the Igri × Triumph segregating population in barley, a strong SD QTL was detected and late flowering is associated with the Igri allele, which is a partial deletion on *HvFT3* (Faure *et al*. 2007). The association of late flowering with the partial deletion of the Igri *HvFT3* parallels the results from the current study in wheat, where deletions of the *TaFT3‐B1* gene result in late flowering (Figs 4b & S3), supporting that the gene could have the same floral promoting function in both species. Another study in barley showed that overexpression of *HvFT3* results in early flowering (Kikuchi *et al*. 2009). A recent report in barley showed that increasing the copy number of *HvFT1*, a relative of *FT3*, accelerated flowering (Nitcher *et a*l. 2013). Taken together, the results from studies in barley and this study suggest that *TaFT3‐B1*, like other *FT* family genes, is a promoter of flowering. Within the scope of this study, we were unfortunately not able to define the extent of the deletion that includes *TaFT3‐B1*, a region that may include other candidate genes. However, given that the single nucleotide polymorphism, which changes a conserved amino acid of *TaFT3‐B1* in Spark, results in a similar phenotype supports *TaFT3‐B1* strongly as the candidate gene underlying the 1BL QTL.

In the case of the discussed *TaFT3‐B1* SNP or the *TaFT3‐B1*deletion, a recessive loss‐of‐function mutation should be the outcome. One would expect the *TaFT3‐A1* and *TaFT3‐D1* copies to compensate for the loss‐of‐function of *TaFT3‐B1*. One possible explanation would be that, particularly because the expression of the *TaFT3‐B1* is lower than that of the other two homoeologues (Fig. 5e,f), the protein encoded by *TaFT3‐B1* has a stronger effect than those of the other two. The *TaFT3‐D1* gene has a conserved amino acid alanine deleted (Fig. S4), which may affect the function of the protein that it encodes.

These results are interesting given that in the Spark × Rialto background, the 1BL QTL is SD specific (Figs 3 & S2a), while in the Charger × Badger background, the QTL is photoperiod independent (Figs 3 & S2b), and in the Avalon × Cadenza background, the QTL is not significant under both SD and LD (Figs 3 & S1). This presents an excellent opportunity to study the gene network in three independent backgrounds by using NILs derived from these crosses. For example, it is possible that a gene or genes that regulate *TaFT3* in LD are mutated either in Charger or in Badger. Not much is known about the genes that regulate *FT3* in temperate cereals, but a recent study in barley showed that *VRNH2* is a repressor of *HvFT3* under LD (Casao *et al*. 2011a).

Recent studies in barley indicate that *HvFT3* (*Ppd‐H2*) allow the adaptation of southern European germplasm to mild winters by promoting early flowering of non‐vernalized plants in SD (Casao *et al*. 2011b). Dubcovsky *et al*. (2006) suggested that wheat was ancestrally a short–long day plant, but artificial selection led to loss of SD regulation. Our work, which has identified two genes that regulate flowering under SD in wheat, supports the suggestion by Dubcovsky *et al*. (2006) that wheat could have ancestrally had short–long day dual regulation. Mutations at *TaTOE1‐B1* and *TaFT3‐B1* could account for the difference in sensitivity to reduced day length between winter wheat cultivar Rialto relative to Spark, which we showed in an earlier report Zikhali *et al*. (2014). Rialto has a functional *TaFT3‐B1* allele and a mutant *TaTOE1‐B1* allele, while Spark has a mutant *TaFT3‐B1* allele and a functional *TaTOE1‐B1* allele. Rialto and Spark would be predicted to be early and late flowering under SD, respectively, and our results suggest that this is the case (Table 1 & Figs 2 & 4).

Here, we also offer support at the expression level for *TaTOE1* and *TaFT3* homoeologues that these two genes have an effect on flowering time under SD where they function to repress and promote flowering, respectively (Fig. 5). The A. thaliana *RAP2.7* gene, a homologue of *TaTOE1*, is a floral repressor, and overexpression of this gene delays flowering (Aukerman and Sakai 2003). Our results show that *TaTOE1* has a similar function in wheat as in A. thaliana where mutants at this gene are early flowering (Fig. 2 & Table 1). We also show that when *TaTOE1* expression is high, *TaFT1* expression is low and vice versa (Fig. 5), suggesting that *TaTOE1* is a repressor of *TaFT1* as suggested by the temperate cereals flowering time model (Higgins *et al*. 2010). High expression detected for the *TaFT1‐B* copy relative of the other two homoeologues was consistent with a study by Lv *et al*. (2014). In barley, mutants at *HvFT3* (*PpdH2*) delay flowering (Faure *et al*. 2007) and our results for *TaFT3‐B1* suggest that the gene has a similar function in wheat as in barley. While *TaFT3‐B1* is a good candidate for *Ppd‐B2*, we did not use that name because another locus on chromosome 7BS has already been named *Ppd‐B2* (Khlestkina *et al*. 2008).

We also offer evidence from two diversity panels that both *TaTOE1‐B1* and *TaFT3‐B1* have significant effect on flowering time (Fig. 6). Our results suggest that *TaFT3‐B1* has a stronger effect than *TaTOE1‐B1* (Fig. 6). Our work will add to the growing body of knowledge on flowering time in cereals and will help in modelling of flowering time in wheat, a strategic food security crop. Understanding of flowering in cereals is crucial to global food security given that the top five important cereal crops: wheat, maize rice, barley and sorghum, need optimum flowering time to achieve the high yields needed for global food security.

The identification of these two genes on group 1 chromosomes is consistent with Law *et al*. (1998), who proposed that there was more than one flowering time gene on the group 1 homologous chromosomes. Furthermore, Law *et al*. (1998) suggested that one of the genes on group 1 was on the short arm and that among the many genes on group 1 chromosomes, one of them was a suppressor of flowering time. Consistent with the multiple gene hypothesis on group 1 chromosomes by Law *et al*. (1998), this study identified a suppressor of flowering time on the short arm of 1B (*TaTOE1‐B1*) and a flowering time promoter on 1BL (*TaFT3‐B1*) a likely homoeologue of the gene responsible for the QTL identified on 1AL (Kuchel *et al*. 2006); in addition to the floral repressor on the distal end of 1DL, we identified in our earlier studies (Zikhali *et al*. 2015; Zikhali *et al*. 2014). The two genes *TaFT3‐B1 and TaTOE1‐B1* that we have identified in this study will provide additional control for wheat breeders in the quest to breed better, adapted and more resilient cultivars.

## Accession Numbers

Sequence data derived from this paper can be found in the Genbank sequence data base under the following accession numbers:

1. Triticum aestivum flowering locus T3‐B1 (TaFT3‐B1) Spark (KJ711538), Rialto (KJ711539), Badger (KJ711540), Cadenza (KJ711541) and Malacca (KJ711548).
2. Triticum aestivum flowering locus T3‐A1 (TaFT3‐A1) Claire (KJ711527), Hereward (KJ711528), Malacca (KJ711531), Charger (KJ711532), Badger (KJ711533), Cadenza (KJ711534), Avalon (KJ711535), Spark (KJ711536), Rialto (KJ711537), Trident (KT824056) and Molineux (KT824057).
3. Triticum aestivum flowering locus T3‐D1 (TaFT3‐D1) Spark (KJ661739), Rialto (KJ661740), Cadenza (KJ676791), Avalon (KJ676792), Badger (KJ676793), Charger (KJ676794), Malacca (KJ676795), Hereward (KJ676796) and Claire (KJ676797).
4. Triticum aestivum WUSCHEL‐like‐B1 (TaWUSCHELL‐B1) Avalon (KT285832), Cadenza (KT285833), Badger (KT285834), Charger (KT285835), Claire (KT285836) and Spark (KT285837).
5. Triticum aestivum SENSITIVITY TO RED LIGHT REDUCED 1‐B1 (TaSRR1‐B1) Avalon (KT285838), Cadenza (KT285839) and Charger (KT285840).
6. Triticum aestivum TARGET OF EAT1‐B1 (TaTOE1‐B1) Avalon (KT439183), Cadenza (KT439184), Charger (KT439185), Badger (KT439186) and Spark (KT439187).

## Supporting information


**Figure S1.** Short‐day‐specific 1BS Heading date QTL of Avalon × Cadenza DH population
**Figure S2.** Spark × Rialto 1BL short‐day‐specific heading date QTL and Charger × Badger 1BL photoperiod‐independent heading date QTL
**Figure S3.** Genotyping of the Charger × Badger, Avalon × Cadenza and Spark × Rialto DH populations with the mutations at *TaFT3‐B1*
**Figure S4.** Deletion of a conserved amino acid alanine in *TaFT3‐D1* copy

**Table S1.** The 21 syntenic B. distachyon genes used to define the gens in the 1BS QTL interval peak.
**Table S2.** The genome‐specific primer sequences used to sequence the genes *TaFT3‐A1*, *TaFT3‐B1*, *TaFT3‐D1*, *TaSRR1‐B1*, *TaWUSCHELL‐B1* and *TaTOE1‐B1*
**Table S3.** The primer combinations for *TaFT3‐B1* KASP marker
**Table S4.** The hierarchical STUCTURE analysis of the Watkins population based on SSR data.

## References

[pce13018-bib-1001] Allen A.M. , Barker G.L.A. , Berry S.T. , Coghill J.A. , Gwilliam R. , Kirby S. , … Edwards K.J. (2011) Transcript‐specific, single‐nucleotide polymorphism discovery and linkage analysis in hexaploid bread wheat (*Triticum aestivum* L.). Plant Biotechnology Journal 9, 1086–1099. 21627760 10.1111/j.1467-7652.2011.00628.x

[pce13018-bib-0001] Aukerman M.J. & Sakai H. (2003) Regulation of flowering time and floral organ identity by microRNA and its APETALA2‐like target genes. The Plant Cell 15, 2730–2741. 14555699 10.1105/tpc.016238 PMC280575

[pce13018-bib-0002] Beales J. , Turner A. , Griffiths S. , Snape J.W. & Laurie D.A. (2007) A pseudo‐response regulator is misexpressed in the photoperiod insensitive Ppd‐D1a mutant of wheat (Triticum aestivum L.). Theoretical and Applied Genetics 115, 721–733. 17634915 10.1007/s00122-007-0603-4

[pce13018-bib-0003] Bradbury P.J. , Zhang Z. , Kroon D.E. , Casstevens T.M. , Ramdoss Y. & Buckler E.S. (2007) TASSEL: software for association mapping of complex traits in diverse samples. Bioinformatics 23, 2633–2635. 17586829 10.1093/bioinformatics/btm308

[pce13018-bib-0004] Casao C.M. , Igartua E. , Karsai I. , Lasa J.M. , Gracia P.M. & Casas A.M. (2011a) Expression analysis of vernalization and day‐length response genes in barley (Hordeum vulgare L.) indicates that VRNH2 is a repressor of PPDH2 (HvFT3) under long days. Journal of Experimental Botany 6, 1939–1949. 10.1093/jxb/erq382 PMC3060678 21131547

[pce13018-bib-0005] Casao M.C. , Karsai I. , Igartua E. , Gracia M.P. , Veisz O. & Casas A.M. (2011b) Adaptation of barley to mild winters: a role for PPD‐H2. BMC Plant Biology 11, 164. 22098798 10.1186/1471-2229-11-164 PMC3226555

[pce13018-bib-0006] Chen A. & Dubcovsky J. (2012) Wheat TILLING mutants show that the vernalization gene VRN1 down‐regulates the flowering repressor VRN2 in leaves but is not essential for flowering. PLoS Genetics 8, e1003134. https://doi.org/10.1371/journal.pgen.1003134. 10.1371/journal.pgen.1003134 PMC3521655 23271982

[pce13018-bib-0007] Danilevskaya O.N. , Meng X. , Hou Z. , Ananiev E.V. & Simmons C.R. (2008) A genomic and expression compendium of the expanded PEBP gene family from maize. Plant Physiology 146, 250–264. 17993543 10.1104/pp.107.109538 PMC2230559

[pce13018-bib-0008] Dı'az A. , Zikhali M. , Turner A.S. , Isaac P. , & Laurie D.A. (2012) Copy number variation affecting the photoperiod‐B1 and vernalization‐A1 genes is associated with altered flowering time in wheat (Triticum aestivum). PloS One 7, e33234. https://doi.org/10.1371/journal.pone. 0033234. 10.1371/journal.pone.0033234 PMC3310869 22457747

[pce13018-bib-0009] Distelfeld A. , Li C. & Dubcovsky J. (2009) Regulation of flowering in temperate cereals. Current Opinion in Plant Biology 12, 1–7. 19195924 10.1016/j.pbi.2008.12.010

[pce13018-bib-0010] Dong Z. , Danilevskaya O. , Abadie T. , Messina C. , Coles N. & Cooper M. (2012) A gene regulatory network model for floral transition of the shoot apex in maize and its dynamic modeling. PloS One 7, e43450. https://doi.org/10.1371/journal.pone.0043450. 10.1371/journal.pone.0043450 PMC3422250 22912876

[pce13018-bib-0011] Dubcovsky J. , Loukoianov A. , Fu D. , Valarik M. , Sanchez A. & Yan L. (2006) Effect of photoperiod on the regulation of wheat vernalization genes VRN1 and VRN2. Plant Molecular Biology 60, 469–480. 16525885 10.1007/s11103-005-4814-2 PMC4739792

[pce13018-bib-0012] Faure S. , Higgins J. , Turner A. & Laurie D.A. (2007) The FLOWERING LOCUS T‐like gene family in barley (Hordeum vulgare). Genetics 176, 599–609. 17339225 10.1534/genetics.106.069500 PMC1893030

[pce13018-bib-0014] Herndl M. , White J.W. , Hunt L.A. , Graeff S. & Claupein W. (2008) Field‐based evaluation of vernalization requirement, photoperiod response and earliness per se in bread wheat (Triticum aestivum L). Field Crops Research 105, 193–201.

[pce13018-bib-0015] Higgins J.A. , Bailey P.C. & Laurie D.A. (2010) Comparative genomics of flowering time pathways using Brachypodium distachyon as a model for the temperate grasses. PloS One 5, e10065. https://doi.org/10.1371/journal.pone.0010065. 10.1371/journal.pone.0010065 PMC2856676 20419097

[pce13018-bib-0016] Jung J.H. , Seo Y.H. , Seo P.J. , Reyes J.L. , Yun J. , Chua N.H. & Park C.M. (2007) The GIGANTEA regulated microRNA172 mediates photoperiodic flowering independent of CONSTANS in Arabidopsis. Plant Cell 19, 2736–2748. 17890372 10.1105/tpc.107.054528 PMC2048707

[pce13018-bib-0017] Khlestkina E.K. , Giura A. , Roder M.S. & Borner A. (2008) A new gene controlling the flowering response to photoperiod in wheat. Euphytica 165, 579–585.

[pce13018-bib-0018] Kikuchi R. , Kawahigashi H. , Ando T. , Tonooka T. & Handa H. (2009) Molecular and functional characterization of PEBP genes in barley reveal the diversification of their roles in flowering. Plant Physiology 149, 1341–1353. 19168644 10.1104/pp.108.132134 PMC2649388

[pce13018-bib-0019] Kuchel H. , Hollamby G.J. , Langridge P. , Williams K.J. & Jefferies S.P. (2006) Identification of genetic loci associated with ear‐emergence in bread wheat. Theoretical and Applied Genetics 113, 1103–1112. 16896709 10.1007/s00122-006-0370-7

[pce13018-bib-0020] Kumar S. , Sharma V. , Chaudhary S. , Tyagi A. , Mishra P. , Priyadarshini A. & Singh A. (2012) Genetics of flowering time in bread wheat Triticum aestivum: complementary interaction between vernalization‐insensitive and photoperiod‐insensitive mutations imparts very early flowering habit to spring wheat. Journal of Genetics 91, 33–47. 22546824 10.1007/s12041-012-0149-3

[pce13018-bib-0021] Laurie D.A. , Pratchett N. , Bezant J.H. & Snape J.W. (1995) RFLP mapping of five major genes and eight quantitative trait loci controlling flowering time in a Winter × Spring barley Hordeum vulgare L. cross. Genome 38, 575–585. 18470191 10.1139/g95-074

[pce13018-bib-0022] Lauter N. , Kampani A. , Carlson S. , Goebel M. & Moose S.P. (2005) microRNA172 down‐regulates glossy15 to promote vegetative phase change in maize. Proceedings of the National Academy of Sciences of the United States of America 102, 9412–9417. 15958531 10.1073/pnas.0503927102 PMC1166634

[pce13018-bib-0023] Laux T. , Klaus F.X. , Mayer K.F.X. , Berger J. & Gerd J.G. (1996) The WUSCHEL gene is required for shoot and floral meristem integrity in Arabidopsis. Development 122, 87–96. 8565856 10.1242/dev.122.1.87

[pce13018-bib-0024] Law C.N. , Suarez E. , Miller J.R. & Worland A.J. (1998) The influence of the group 1 chromosomes of wheat on ear‐emergence times and their involvement with vernalization and day length. Heredity 80, 83–91.

[pce13018-bib-0025] Li C.X. , Distelfeld A. , Comis A. & Dubcovsky J. (2011) Wheat flowering repressor VRN2 and promoter CO_2_ compete for interactions with NUCLEAR FACTOR‐Y complexes. The Plant Journal 67, 763–773. 21554456 10.1111/j.1365-313X.2011.04630.x PMC4765905

[pce13018-bib-0026] Lv B. , Nitcher R. , Han X. , Wang S. , Ni F. , et al. (2014) Characterization of FLOWERING LOCUS T1 (FT1) gene in Brachypodium and wheat. PloS One 9, e94171. https://doi.org/10.1371/journal.pone.0094171. 10.1371/journal.pone.0094171 PMC3981775 24718312

[pce13018-bib-0027] Mcintosh R.A. , Yamazaki Y. , Devos K.M. , Dubcovsky J. , Rogers W.J. & Appels R. (2003) Catalogue of gene symbols for wheat In International Wheat Genetics Symposium. (10°, 1–6 September, (2003) Instituto Sperimentale per la Cerealicoltura, Rome, Paestum, Italy), Vol. 4, p. 77.

[pce13018-bib-0028] Milec Z. , Valárik M. , Bartoš J. & Šafář J. (2014) Can a late bloomer become an early bird? Tools for flowering time adjustment. Biotechnology Advances 32, 200–214. 24091290 10.1016/j.biotechadv.2013.09.008

[pce13018-bib-0029] Knight E. , Binnie A. , Draeger T. , Moscou M. , Rey M.‐D. , Sucher J. , … Moore G. (2015) Mapping the ‘breaker’ element of the gametocidal locus proximal to a block of sub‐telomeric heterochromatin on the long arm of chromosome 4Ssh of *Aegilops sharonensis* . Theoretical and Applied Genetics 128, 1049–1059. 25748115 10.1007/s00122-015-2489-x PMC4435904

[pce13018-bib-0030] N'Diaye A. , Haile J.K. , Cory A.T. , Clarke F.R. , Clarke J.M. , Knox R.E. , et al. (2017) Single marker and haplotype‐based association analysis of semolina and pasta colour in elite durum wheat breeding lines using a high‐density consensus map. PloS One 12, e0170941. https://doi.org/10.1371/journal.pone.0170941. 10.1371/journal.pone.0170941 PMC5279799 28135299

[pce13018-bib-0031] Nitcher R. , Disterfield A. , Tan C. , Yan L. & Dubcovsky J. (2013) Increased copy number at the HvFT1 locus is associated with accelerated flowering time in barley. Molecular Genetics and Genomics 288, 261–275. 23591592 10.1007/s00438-013-0746-8 PMC3664738

[pce13018-bib-0032] Okamuro J.K. , Caster B. , Villarroel R. , Montagu M.V. & Jofuku K.D. (1997) The AP2 domain of APETALA2 defines a large new family of DNA binding proteins in Arabidopsis. Proceedings of the National Academy of Sciences of the United States of America 94, 7076–7081. 9192694 10.1073/pnas.94.13.7076 PMC21287

[pce13018-bib-0033] Salvi S. , Sponza G. , Morgante M. , Tomes D. , Niu X. , Fengler K. , … Tuberosa R. (2007) Conserved non coding genomic sequences associated with flowering‐time quantitative trait locus in maize. Proceedings of the National Academy of Sciences of the United States of America 104, 11 376–11 381. 10.1073/pnas.0704145104 PMC2040906 17595297

[pce13018-bib-0034] Shaw L.M. , Turner A.S. & Laurie D.A. (2012) The impact of photoperiod insensitive Ppd‐1a mutations on the photoperiod pathway across the three genomes of hexaploid wheat (Triticum aestivum). The Plant Journal 71, 71–84. 22372488 10.1111/j.1365-313X.2012.04971.x

[pce13018-bib-0035] Shrestha R. , Gómez‐Ariza J. , Brambilla V. & Fornara F. (2014) Molecular control of seasonal flowering in rice. Arabidopsis and temperate cereals. Annals of Botany. 114, 1445–1458. 24651369 10.1093/aob/mcu032 PMC4204779

[pce13018-bib-0036] Staiger D. , Allenbach L. , Salathia N. , Fiechter V. , Davi S.J. , Millar A.J. , … Fankhauser C. (2003) The Arabidopsis SRR1 gene mediates phyB signaling and is required for normal circadian clock function. Genes and Development 17, 256–268. 12533513 10.1101/gad.244103 PMC195977

[pce13018-bib-0037] Turner A. , Beales J. , Faure S. , Dunford R.P. & Laurie D.A. (2005) The pseudo‐response regulator Ppd‐H1 provides adaptation to photoperiod in barley. Science 310, 1031–1034. 16284181 10.1126/science.1117619

[pce13018-bib-0038] Wilhelm E.P. , Turner A.S. & Laurie D.A. (2009) Photoperiod insensitive Ppd‐A1a mutations in tetraploid wheat (Triticum durum Desf.). Theoretical and Applied Genetics 118, 285–294. 18839130 10.1007/s00122-008-0898-9

[pce13018-bib-0039] Wingen L.U. , Orford S. , Goram R. , Leverington‐Waite M. , Bilham L. , Patsiou T.S. , … Griffiths S. (2014) Establishing the A. E. Watkins landrace cultivar collection as a resource for systematic gene discovery in bread wheat. Theoretical and Applied Genetics 127, 1831–1842. 24985064 10.1007/s00122-014-2344-5 PMC4110413

[pce13018-bib-0040] Yan L. , Fu D. , Li C. , Blechl A. , Tranquilli G. , Bonafede M. , … Dubcovsky J. (2006) The wheat and barley vernalization gene VRN3 is an orthologue of FT. Proceedings of the National Academy of Sciences of the United States of America 103, 19 581–19 586. 10.1073/pnas.0607142103 PMC1748268 17158798

[pce13018-bib-0042] Yan L. , Loukoianov A. , Tranquilli G. , Helguera M. , Fahima T. & Dubcovsky J. (2003) Positional cloning of the wheat vernalization gene VRN1. Proceedings of the National Academy of Sciences of the United States of America 100, 6263–6268. 12730378 10.1073/pnas.0937399100 PMC156360

[pce13018-bib-0043] Zhu Q.H. & Helliwell C.A. (2011) Regulation of flowering time and floral patterning by miR172. Journal of Experimental Botany 62, 487–495. 20952628 10.1093/jxb/erq295

[pce13018-bib-0044] Zadoks J.C. , Chang T.T. & Konzak C.F. (1974) A decimal code for the growth stages of cereals. Weed Research 14, 415–421.

[pce13018-bib-0045] Zhang Z. , Yang X. , Meng L. , Liu F. , Shen C. & Yang W. (2009) Enhanced amplification of GC‐rich DNA with two organic reagents. BioTechniques 47, 775–779. 19852763 10.2144/000113203

[pce13018-bib-0046] Zikhali M. , Leverington‐Waite M. , Fish L. , Simmonds J. , Orford S. , Wingen L.U. , … Griffiths S. (2014) Validation of a 1DL earliness per se (Eps) flowering QTL in bread wheat (Triticum aestivum). Molecular Breeding 34, 1023–2033. 25242885 10.1007/s11032-014-0094-3 PMC4162975

[pce13018-bib-0047] Zikhali M. , Wingen L.U. & Griffiths S. (2015) Delimitation of the Earliness per se D1 (Eps‐D1) flowering gene to a subtelomeric chromosomal deletion in bread wheat (Triticum aestivum). Journal of Experimental Botany 67, 287–299. 26476691 10.1093/jxb/erv458 PMC4682435

