# Supplementary material for: The identification of new candidate genes Triticum aestivum FLOWERING LOCUS T3‐B1 (TaFT3‐B1) and TARGET OF EAT1 (TaTOE1‐B1) controlling the short‐day photoperiod response in bread wheat
Source: Plant Cell Environ. 2017 Aug 17;40(11):2678–90. doi: 10.1111/pce.13018 (PMC5669021; doi:10.1111/pce.13018)
Supplement: Supplementary file 1 — Figure S1. Short‐day‐specific 1BS Heading date QTL of Avalon × Cadenza DH population Figure S2. Spark × Rialto 1BL short‐day‐specific heading date QTL and Charger × Badger 1BL photoperiod‐independent heading date QTL Figure S3. Genotyping of the Charger × Badger, Avalon × Cadenza and Spark × Rialto DH populations with the mutations at TaFT3‐B1 Figure S4. Deletion of a conserved amino acid alanine in TaFT3‐D1 copy [file PCE-40-2678-s001.docx]

**Supporting material**


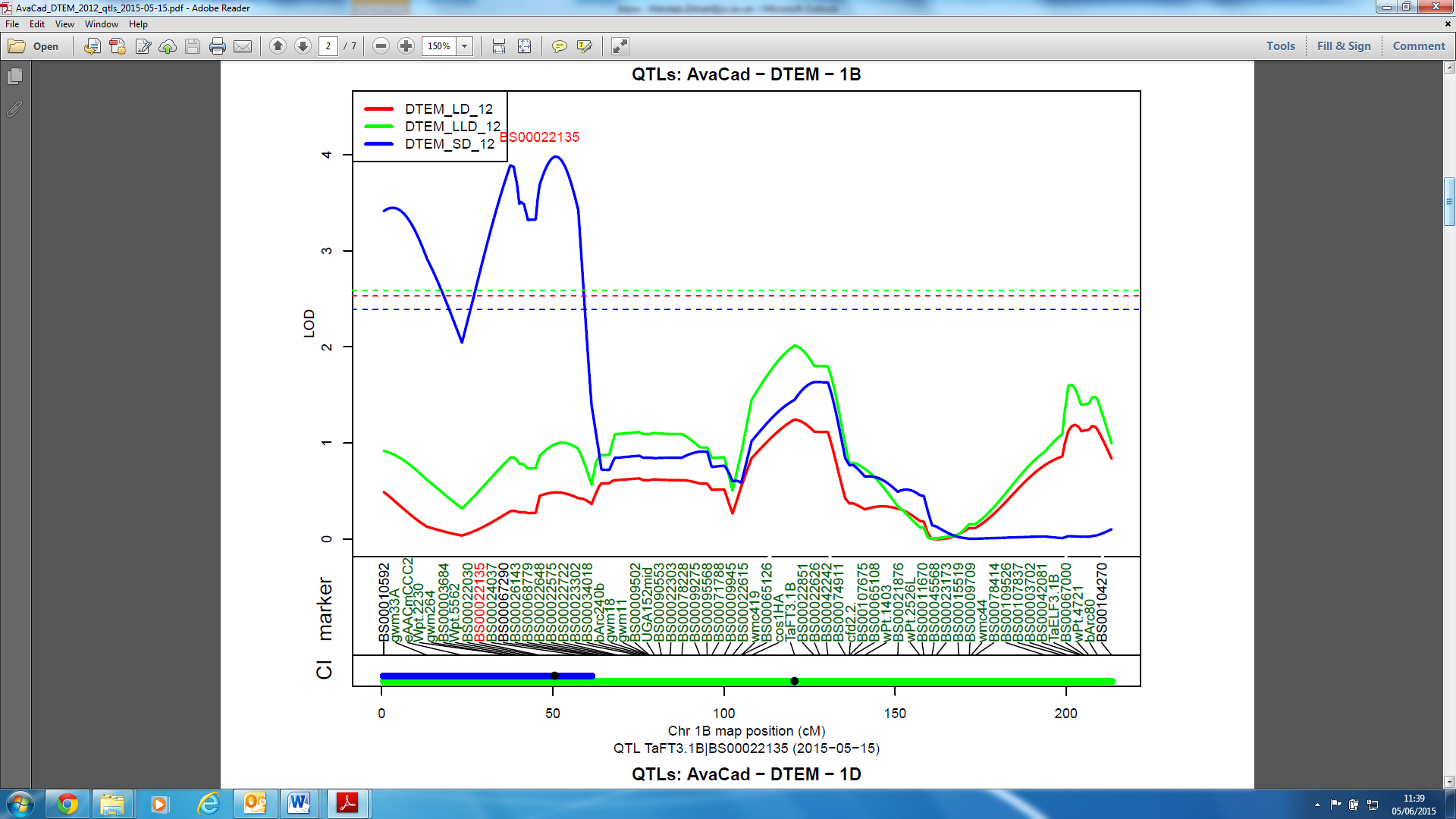


Fig. S1 Heading date QTL of Avalon X Cadenza DH population vernalized at 7-10°C for 8 weeks in short days (SD) 10 hrs light and 14hrs dark and then grown in short days (SD) 10 hrs light, long days (LD) 16 hrs light and very long days (LLD) 20 hrs light.

**
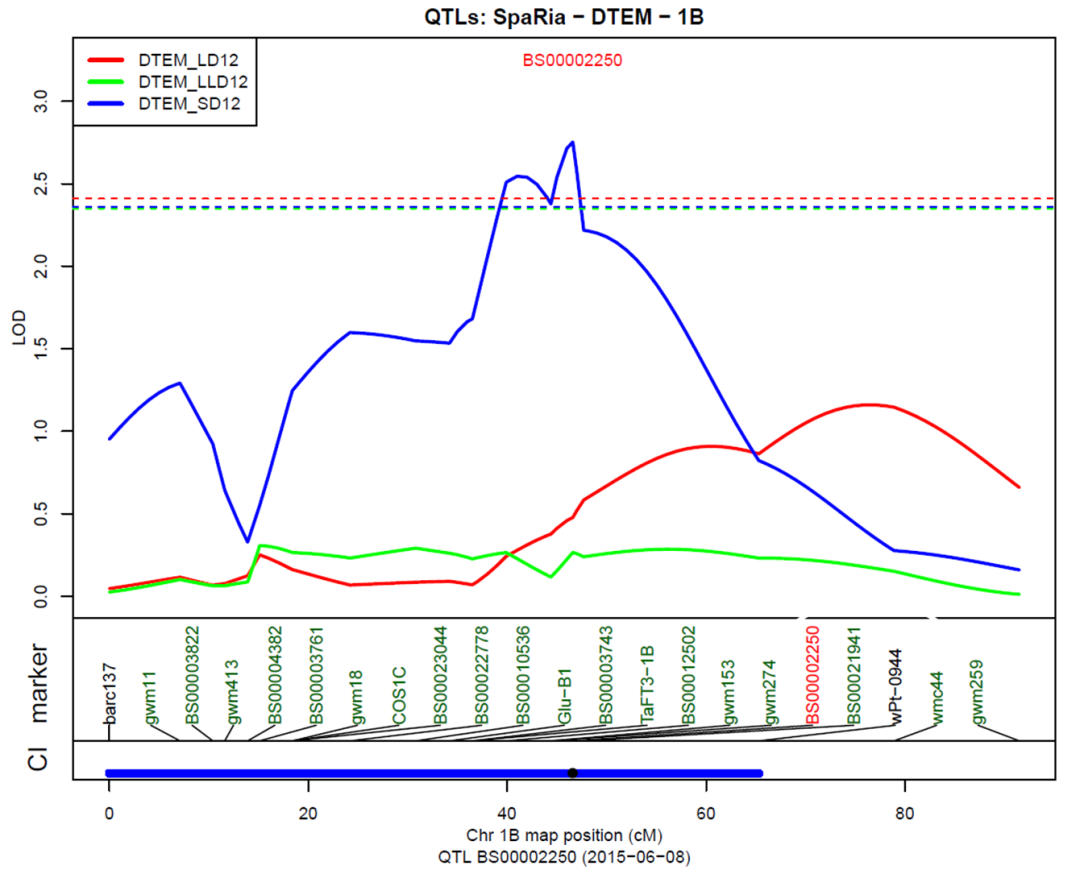
**

A


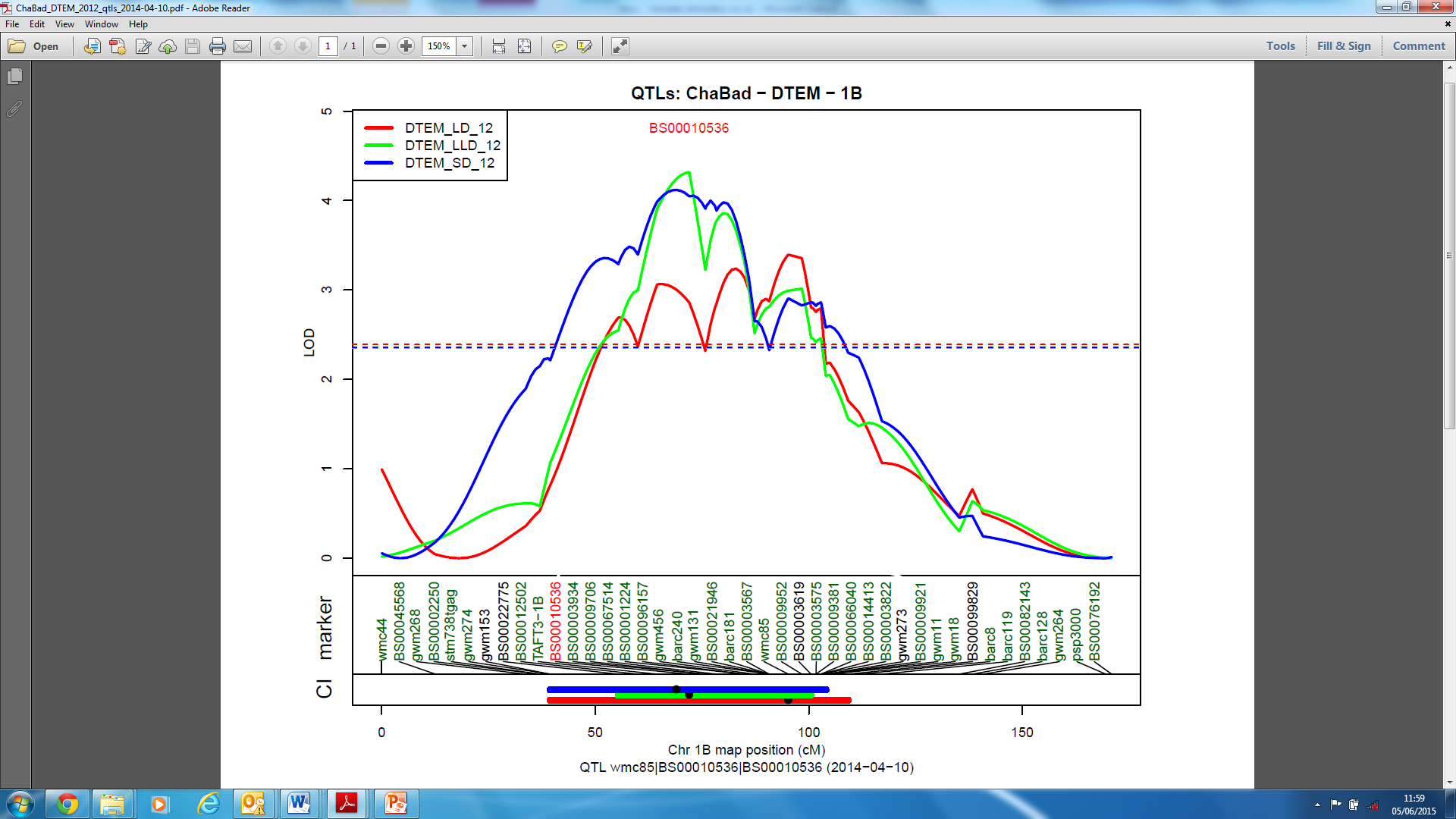


B

Fig. S2 Days to ear emergence QTL of Spark X Rialto (A) and Charger X Badger (B) DH populations vernalized at 7-10°C for 8 weeks and then grown in short days (SD) 10 hrs light, long days (LD) 16 hrs light and very long days (LLD) 20 hrs light. Unlike Spark X Rialto (A), the QTL was detected in all the tested photoperiod treatments for Charger X Badger (B).


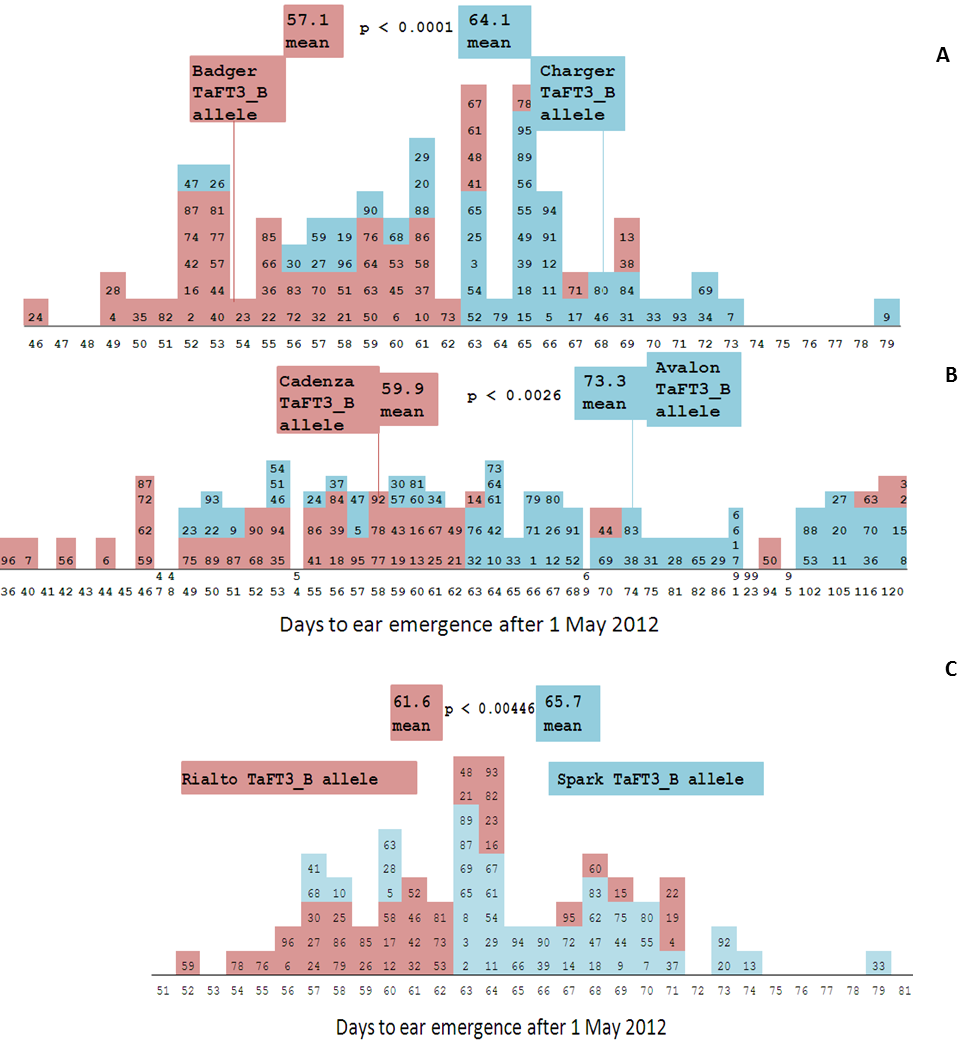


Fig. S3 Days to ear emergence of Charger X Badger (A), Avalon X Cadenza (B) and Spark X Rialto (C) DH populations grown in short days (10hrs light and 14 hrs light) and then scored for the presence (brown highlighted numbers) or absence due to deletion (sky blue highlighted numbers) of the *TaFT3-B1* gene. The Spark X Rialto DH population (C) was scored using the KASP marker that scores the SNP that changes a conserved glycine (Rialto wild type) to serine (Spark, mutant) in the *TaFT3*-*B1* gene. Both mutation of *TaFT3-B1* are likely to delay flowering under short days.

Fig. S4 Deletion (-) of the conserved amino acid alanine (A) in the D genome copy of the *TaFT3* gene.
