# Supplementary material for: The identification of new candidate genes Triticum aestivum FLOWERING LOCUS T3‐B1 (TaFT3‐B1) and TARGET OF EAT1 (TaTOE1‐B1) controlling the short‐day photoperiod response in bread wheat
Source: Plant Cell Environ. 2017 Aug 17;40(11):2678–90. doi: 10.1111/pce.13018 (PMC5669021; doi:10.1111/pce.13018)
Supplement: Supplementary file 2 — Table S1. The 21 syntenic B. distachyon genes used to define the gens in the 1BS QTL interval peak. Table S2. The genome‐specific primer sequences used to sequence the genes TaFT3‐A1, TaFT3‐B1, TaFT3‐D1, TaSRR1‐B1, TaWUSCHELL‐B1 and TaTOE1‐B1 Table S3. The primer combinations for TaFT3‐B1 KASP marker Table S4. The hierarchical STUCTURE analysis of the Watkins population based on SSR data. [file PCE-40-2678-s002.docx]

Table S1 Primers sequences used to amplify the A, B and D gene copies of *TaFT3*, *TaTOE1-B1*, *TaSRR1-B1* and *TaWUSHELL-B1.*

| marker/gene name | Primer sequence 5'-3' | Tm^α^ |
| --- | --- | --- |
| *TaFT3-A1*_5UTR_F1 | TCCGCAGGTAGTTCAGAAATC | 63.1 |
| *TaFT3-A1*_5UTR_R1 | CTCTTCTACTTATTGCCGAAAAAA | 61.4 |
| *TaFT3-A1*_5UTR_F2 | GAAATACTAAAATACATGATGGCCTA | 60.1 |
| *TaFT3-A1*_5UTR_R2 | GCTTAGGGTCATTATTTTATTCAAAA | 61.2 |
| *TaFT3-A1*_5UTR_F3 | TGTCACTGTTGTTTACAAAGTGAA | 61.1 |
| *TaFT3-A1*_5UTR_R3 | GCTGTGGTATACTGGGACG | 60.6 |
| *TaFT3-A1*_5UTR_F4 | AAATACAAGGGAAAGTACTTAATACCAG | 60.7 |
| *TaFT3-A1*_5UTR_R4 | CCAGGACTAGCCTATTGTTGTAA | 60.9 |
| *TaFT3-A1*_F1 | ACAATACAAGTAGTTCGTGCAAGTAGG | 64.11 |
| *TaFT3-A1*_F2 | GCTAGGCACAATAACAGACAAAGG | 64.58 |
| *TaFT3-A1*_F3 | GACAAAGGCTAAGGCTGTTAAC | 60.67 |
| *TaFT3-A1*_F4 | TCCTATTTAAGTTGCCCTGATTAC | 61.03 |
| *TaFT3-A1*_F5 | CAAATGACTCCATACAAGTTAATTCT | 60.19 |
| *TaFT3-A1*_R1 | AAAGCTGGCACCAGTTGTTG | 65.11 |
| *TaFT3-A1*_R2 | CCCTAATATTAATTAGTTTAGAGTTTCAAA | 59.38 |
| *TaFT3-A1*_R3 | ATTATTTGTGATGCAACACTACG | 60.33 |
| *TaFT3-A1*_R4 | AGATGATACAGGGTATTCTATATGCTA | 59 |
| *TaFT3-A1*_R5 | TTCCCACAAGAATACTCTCCG | 62.86 |
|  |  |  |
| *TaFT3-B1*_5UTR_F1 | GTCATCAAATGAAAGCGCGTC | 66.65 |
| *TaFT3-B1*_5UTR_R1 | CTGATATTGCTTATTTAAGGCAATC | 60.66 |
| *TaFT3-B1*_5UTR_F2 | AAGTTTCTATAAAACTTTCCATATGTCATAC | 60.85 |
| *TaFT3-B1*_5UTR_R2 | TGCCAAGTTGCAACGC | 63.52 |
| *TaFT3-B1*_5UTR_F3 | CTAAAGCCAACACAAAAGCAGATTG | 66.37 |
| *TaFT3-B1*_5UTR_R3 | GCGATAGTTAGTGCATTCCTTAGA | 62.24 |
| *TaFT3-B1*_5UTR_F4 | ACCCTGTTCCAACATAGAGTAGCGT | 66.6 |
| *TaFT3-B1*_5UTR_R4 | TTGATACTAGCGTCATGTTCTGT | 60.22 |
| *TaFT3-B1*_5UTR_F5 | CATTATTTCCATAGAGGTTGTGATCT | 62.03 |
| *TaFT3-B1*_5UTR_R5 | GAGGACTACCAATTAATAGCCTTGT | 61.37 |
| *TaFT3-B1*_F1 | GTTACGTGTATACAGGTGACACAG | 59.92 |
| *TaFT3-B1*_F2 | GACACAGTTTGTTTCAGGTCTC | 60.21 |
| *TaFT3-B1*_F3 | GCAGACAAGGACAAAGGCTA | 61.94 |
| *TaFT3-B1*_F4 | CGACATCCCTGGAACAACTAGTGG | 69.4 |
| *TaFT3-B1*_F5 | AAGCTAATTCCAATATAGTCCATCA | 60.57 |
| *TaFT3-B1*_F6 | GAAGAGTTTTATGGTACACTCCCTAT | 60.51 |
| *TaFT3-B1*_R1 | ATGACCTGATTTATGCATCTG | 58.99 |
| *TaFT3-B1*_R2 | CAAATGTCATTTTACCCTGTTTAGAT | 61.88 |
| *TaFT3-B1*_R3 | TTAGTCATTTATCTGCGATGTG | 59.23 |
| *TaFT3-B1*_R4 | TTCTATATACTGCACATTATTTGTGGTA | 60.42 |
| *TaFT3-B1*_R5 | GATTGAGATATATTCCATCAAGCTT | 60.21 |
| *TaFT3-B1*_R6 | CAGGTGATTAACTAATGATTGAGATATATT | 60.56 |
|  |  |  |
| *TaFT3-D1*_F1 | GAGTATACAGGTGACGCACG | 60.78 |
| *TaFT3-D1*_F2 | GTAGTTCGTGCAAGTAGTTCCTG | 61.62 |
| *TaFT3-D1*_F3 | AGTTCCTGCATAGACACAACTG | 60.78 |
| *TaFT3-D1*_F4 | CATGAAACTTTAGATGTATGTTCCATAC | 61.46 |
| *TaFT3-D1*_F5 | TCGTTAACACCAAGACATCTTG | 61.51 |
| *TaFT3-D1*_F6 | AGTATGTTTTGCAAATGAAATGG | 61.45 |
| *TaFT3-D1*_R1 | TGTTATTCTTTGTCATGGTTCAAGATG | 65.57 |
| *TaFT3-D1*_R2 | ACAGTGGCCTTGTCTTAATGTT | 61.34 |
| *TaFT3-D1*_R3 | TGGTTCTGGCCTTTCATAAACTAA | 64.31 |
| *TaFT3-D1*_R4 | ATGGTCAGTACTCTGTACTATCTAGTCC | 59.95 |
| *TaFT3-D1*_R5 | AGTACCGCATACAATGGTCAG | 61.38 |
| *TaFT3-D1*_R6 | CATATAATGCTGCATATTTAGGTGAC | 61.25 |
| *TaFT3*-ngs_F1 | AATAAGACAACCCTCATCGC | 60.5 |
| *TaFT3*-ngs_R1 | CTCCCTTAGTGATGGGTGAC | 60.95 |
| *TaFT3*-ngs_F2 | CCCTGGTAAGCTTCTAACTCTAGT | 59.88 |
| *TaFT3*-ngs_R2 | CAAAACCAGTATAAGATTGAATGATATT | 60.26 |
| *TaFT3*-ngs_F3 | CATGTTAAGTTCTTTCCCTCAAA | 60.86 |
| *TaFT3*-ngs_R3 | CTACTCCCCTTGAGAACTTTCTG | 62 |
|  |  |  |
| *TaTOE1-B1*_F1 | GTATCCGCAGGGAATGC | 61.7 |
| *TaTOE1-B1*_R1 | CGGTGTCAGAAACGTGGA | 63.6 |
| *TaTOE1-B1*_F2 | GACCCCGCTTTCTATATATACG | 60.3 |
| *TaTOE1-B1*_R2 | CGCCGAGATCACTTCTCT | 60.8 |
| *TaTOE1-B1*_F3 | AAACGCGTCGGCACTA | 61.7 |
| *TaTOE1-B1*_R3 | GGATCCTCATGTCCGGA | 62.3 |
| *TaTOE1-B1*_F4 | CGAGGAGGAGGCCACT | 61.7 |
| *TaTOE1-B1*_R4 | CGGTGAAATGACATGACTTG | 61.6 |
| *TaTOE1-B1*_F5 | CTTGCTCCTGTTCATCGCTA | 63.1 |
| *TaTOE1-B1*_R5 | TCATCCACCAAACAACACAC | 62 |
| *TaTOE1-B1*_F6 | TGTTCCTCATCCAACCAAATAG | 62.6 |
| *TaTOE1-B1*_R6 | CCTGCACCCAAACATGTC | 62.8 |
| *TaTOE1-B1*_F7 | TCTGATCTGAATCTGAATCTCG | 61 |
| *TaTOE1-B1*_R7 | GCCAAATCACAGATGCAA | 60.9 |
| *TaTOE1-B1*_F8 | GCTGAAGGTAACAACA**C**GATG | 62.1 |
| *TaTOE1-B1*_R8 | GCATAACACGCAAACGATA | 59.8 |
| *TaTOE1-B1*_F9 | TCCTGCCTGACTATGGTTG | 61.3 |
| *TaTOE1-B1*_R9 | CTATACATATCCTACGACTCTGAACTCA | 61.6 |
| *TaTOE1-B1*_F10 | CATAGTCCACCCGTTCCA | 62.1 |
| *TaTOE1-B1*_R10 | ATGCTCATGTAGGATAAACTCAAG | 60.1 |
| *TaTOE1-B1*_F11 | GCGATAGGAATTAGACCAATTG | 61.2 |
| *TaTOE1-B1*_R11 | ATTATGGGACCAATGCTGC | 62.3 |
| *TaTOE1-B1*_F12 | CAACCTGGGCATGGAGT | 62.3 |
| *TaTOE1-B1*_R12 | GAGCCAAATCTTGTACCAGC | 61.3 |
| *TaTOE1-B1*_F12 | CTCGTGATTGAGATTGTTTGA | 60.3 |
| *TaTOE1-B1*_R12 | ATGCTGCGGCACGGA | 67.1 |
| *TaTOE1-B1*_F13 | GATTGATTGGAAGTTGGAGAGAA | 63.1 |
| *TaTOE1-B1*_R13 | CGCAATCCCTGCATTT | 60.8 |
| *TaTOE1-B1*_F14 | CTCCACCTCCGCCGA | 65.3 |
| *TaTOE1-B1*_R14 | TCACATCACAATTATGTACTCCTG | 60.4 |
| *TaTOE1-B1*_F15 | GTCCGCCGCCGATGT | 67.7 |
| *TaTOE1-B1*_R15 | GGTTGGTGGCTTTGCC | 62.9 |
| *TaTOE1-B1*_F16 | CGCAGTTTGCCATTTGG | 64 |
| *TaTOE1-B1*_R16 | GAGGAGGGTGGCTCACA | 62.7 |
| *TaTOE1-B1*_F17 | GGTTTGCCATTCCTTTTCT | 60.5 |
| *TaTOE1-B1*_R17 | TCATGAATCCAATCCACTACC | 61.2 |
| *TaTOE1-B1*_F18 | ATTTCTGCCAATTTTCTTGAG | 59.8 |
| *TaTOE1-B1*_R18 | CAGATGGTGAGATGAGCTGAC | 62.6 |
| *TaTOE1-B1*_F19 | GATTGATTGGAAGTTGGAGAGAA | 63.1 |
| *TaTOE1-B1*_R19 | GGCACGGTGAAATGACAT | 62.1 |
| *TaTOE1-B1*_F20 | GTTGATTGATTGGAAGTTGGAG | 62.2 |
| *TaTOE1-B1*_R20 | CTAATGCACTCTGCATCTGAACT | 62.3 |
| *TaTOE1-B1*_F21 | CGGAGCTGCTCAGTGAG | 61.2 |
| *TaTOE1-B1*_R21 | GACCATCTCAGGGGGC | 61.8 |
| *TaTOE1-B1*_F22 | TGCTGAAGGTAACAACA**T**GATG | 62.5 |
| *TaTOE1-B1*_R22 | GCATAACACGCAAACGATA | 59.8 |
| *TaTOE1-B1*_F23 | GCAGTATGATGCCCTATCGTAG | 62.3 |
| *TaTOE1-B1*_F24 | CTTCATAGGAACTTCTACAGCTATTGTCT | 63 |
|  |  |  |
| *TaSRR1-B1_*F1 | GAAGAACGGACAAGGCTC | 60 |
| *TaSRR1-B1_R1* | GACGCCGAGGAGGGAT | 64.1 |
| *TaSRR1-B1_*F2 | ATCTGGACCGTCGAGACTC | 60.2 |
| *TaSRR1-B1_*R2 | GAGGACGGGGTCGAAA | 62.4 |
| *TaSRR1-B1_*F3 | CGTCGAGACTCGCCATC | 63.5 |
| *TaSRR1-B1_*R3 | GCATGTAGAAGAGGGTGGA | 60.3 |
| *TaSRR1-B1_*F4 | CCTCCATCCACCACCTC | 61.7 |
| *TaSRR1-B1_*R4 | TCCGCCTGCAGAGCA | 64.8 |
| *TaSRR1-B1_*F5 | GCTGTCGCCACTGCT | 59.8 |
| *TaSRR1-B1_*R5 | GCCCTGCCTTTAGAACG | 61 |
| *TaSRR1-B1_*F6 | CCCCGATCCCGTGGT | 66 |
| *TaSRR1-B1_*R6 | CAAAGCTTGGGAACTTATCTCCT | 63.4 |
| *TaSRR1-B1_*F7 | GCCGCGTGGAGGAGT | 64.1 |
| *TaSRR1-B1_*R7 | CAGTGATAAATTATGCTCAGATGCTT | 63.2 |
| *TaSRR1-B1_*F8 | CAGCTTCCGGCGGTAT | 62.5 |
| *TaSRR1-B1_*R8 | CATTAAGATGCCAACTCTGAAC | 60.2 |
| *TaSRR1-B1_*F9 | CAAGGCCAATCTCGTTCTA | 60.4 |
| *TaSRR1-B1_*R9 | GAATTACAGTGTTAAATGCCAACA | 61.5 |
|  |  |  |
| *TaWUSCHELL-B1_*F1 | CACTGGAGAGTGTCGCCT | 61.8 |
| *TaWUSCHELL-B1_R1* | ATGCGGCCGTAGTGGA | 64.2 |
| *TaWUSCHELL-B1_*F2 | GCTTGCCACACAGGGG | 64.4 |
| *TaWUSCHELL-B1_*R2 | GGCGGCGTAGTAGTGG | 60.5 |
| *TaWUSCHELL-B1_*F3 | CCGTCCGCAGTCCACT | 63.9 |
| *TaWUSCHELL-B1_*R3 | AGCAGCGGAGGGGGA | 66.1 |
| *TaWUSCHELL-B1_*F4 | CCACTCCACCAATAGATCGAG | 63.4 |
| *TaWUSCHELL-B1_*R4 | AGCAGCAGCAGCAGCA | 63.9 |
| *TaWUSCHELL-B1_*F5 | CACGGCGCACCTGGT | 66.4 |
| *TaWUSCHELL-B1_*R5 | GGACGACAAGGTAGCTTAGGTTT | 63.4 |
| *TaWUSCHELL-B1_*F6 | CTCCTCTCCTGCGCC | 60.9 |
| *TaWUSCHELL-B1_*R6 | CCCAGCCTCCCCAGT | 62.5 |
| *TaWUSCHELL-B1_*F7 | TGCTGCTGCTGCTGCT | 63.9 |
| *TaWUSCHELL-B1_*R7 | CTGATCTTCCGTCGCCA | 63.9 |
| *TaWUSCHELL-B1_*F8 | GTCCTTCCATCACCAGGTACA | 63.8 |
| *TaWUSCHELL-B1_*R8 | TGCTCTACCTGCGTACGGT | 63.5 |
| *TaWUSCHELL-B1_*F9 | CTGCTCATGCTCATGCC | 61.9 |
| *TaWUSCHELL-B1_*R9 | CCACACTCCTACTACACCACG | 62.1 |
| *TaWUSCHELL-B1_*F10 | GGATCGCCGGAGTACTCA | 64 |
| *TaWUSCHELL-B1_R1*0 | GCCGCCAACCTACCC | 62.9 |

^α^ = melting temperature D genome specific primer. F and R = forward and reverse primers

Table S2 The 21 names and functions of the syntenous *B. distachyon* genes used to define the genes in the 1BS QTL interval.

| *B. distachyon*  Chromosome 2  Gene number | Match with  *T. aestivum*  Group1S | Gene name and known or predicted function |
| --- | --- | --- |
| *Bradi2g37640* | **yes** | *serine/arginine-rich splicing factor RS2Z33* **(matche*s* KASP marker **XBS00022135*)**  Plays a role in preventing exon skipping, ensuring the accuracy of splicing and regulating alternative splicing) |
| ***Bradi2g37650*** | **yes** | Predicted: *Brachypodium distachyon* ***putative WUSCHEL-related homeobox 2***  The ^α^***WUSCHEL*** gene is needed for the integrity of the shoot and floral meristem in *Arabidopsis thaliana* |
| *Bradi2g37660* | No | Predicted*: Brachypodium distachyon UPF0481 protein At3g47200-like* |
| *Bradi2g37670* | No | Predicted: Brachypodium distachyon *UPF0481 protein At3g47200-like* |
| *Bradi2g37680* | **yes** | Predicted: *Brachypodium distachyon* UPF0014 membrane protein *STAR2*  Associates with STAR2 to form a functional transmembrane ABC transporter required for detoxification of aluminum (Al) in roots. Can specifically transport UDP-glucose |
| *Bradi2g37690* | **yes** | Predicted: *Brachypodium distachyon* glycine-rich protein A3 |
| *Bradi2g37700* | **yes** | Predicted: *Brachypodium distachyon* glycine-rich protein A3-like |
| *Bradi2g37710* | **yes** | uncharacterised |
| *Bradi2g37720* | **yes** | Predicted: *Brachypodium distachyon* WEB family protein At5g16730, chloroplastic-like |
| ***Bradi2g37730*** | **yes** | Predicted: *Brachypodium distachyon protein* ***SENSITIVITY TO RED LIGHT REDUCED 1* (**^β^***SRR1*)***.*  Probable regulator involved in a circadian clock input pathway, which is required for normal oscillator function. Regulates the expression of clock-regulated genes such as *CCA1* and *TOC1*. Involved in both the *phytochrome B* (*PHYB*) and *PHYB*-independent signaling pathways. |
| *Bradi2g37740* | **yes** | uncharacterised |
| *Bradi2g37750* | **yes** | Predicted: *Brachypodium distachyon 125 kDa kinesin-related protein* |
| *Bradi2g37760* | **yes** | Predicted: *Brachypodium distachyon ribonuclease II, chloroplastic/mitochondrial* |
| *Bradi2g37770* | **yes** | uncharacterised |
| *Bradi2g37780* | **yes** | uncharacterised |
| *Bradi2g37790* | **yes** | Predicted: *Brachypodium distachyon transcription factor IIIA-like* |
| ***Bradi2g37800*** | **yes** | Predicted: ***Brachypodium distachyon floral homeotic protein APETALA 2***  *Arabidopsis thaliana* *^µ^RAP2.7 and Zea mays* *RAP2.7* (*^γ^ZmRaP2.7*)  *The RAP2.7* is a floral repressor in *Arabidopsis thaliana* and regulates flowering by repressing *FT* in the ageing pathway.  *ZmRAP2.7* is also a repressor of flowering |
| *Bradi2g37810* | **yes** | Predicted: *Brachypodium distachyon 50S ribosomal protein L10, chloroplastic-like* |
| *Bradi2g37820* |  |  |
| *Bradi2g37830* | No | *uncharacterised* |
| *Bradi2g37840* | **Yes** | Predicted: *Brachypodium distachyon endochitinase A-like* **(matches KASP marker** ^ψ^***XBS00099829*)** |

*^ψ^Allen e*t al*., 2011, ^α^Laux *et al*., 1996; ^β^Staiger *et al*., 2002; *^γ^*Higgins *et al*., 2010

Table S3 KASP assay primer sequences for the genes *TaFT3* –*B1* and *TaTOE1-B1*.

| marker/gene name | Primer sequence 5'-3' | Tm^α^ |
| --- | --- | --- |
|  |  |  |
| *TaFT3-B1* Kasp1-F | gaaggtgaccaagttcatgctAACAACTAGTGGCAGCTTC**A** | 60.07 |
| *TaFT3-B1* Kasp1-V | gaaggtcggagtcaacggattACAACTAGTGGCAGCTTC**G** | 60.64 |
| *TaFT3-B1* Kasp1-C | CATGACCTGATTTATGCATCTG | 61.85 |
|  |  |  |
|  |  |  |
| *TaTOE1-B1*_KASP1_F | gaaggtgaccaagttcatgctCTCCACCTCCGCCG**A** | 85.6 |
| *TaTOE1-B1*_KASP1_V | gaaggtcggagtcaacggattCTCCACCTCCGCCG**C** | 87.5 |
| *TaTOE1-B1*_KASP1_C | CGGGGAACAGCTCTTGG | 64.6 |
|  |  |  |
| *TaTOE1-B1*_KASP2_F | gaaggtgaccaagttcatgctGCTGAAGGTAACAACA**C**GATG | 57.9 |
| *TaTOE1-B1*_KASP2_V | gaaggtcggagtcaacggattTGCTGAAGGTAACAACA**T**GATG | 63.4 |
| *TaTOE1-B1*_KASP2_C | GCCAAATCACAGATGCAA | 64.7 |

^α^ = melting temperature, F = fam and V = vic fluorescent tails, *TaFT3-B1 =* B genome copy of TaFT3, C = common primer. The red and green highlight denotes the fam and vic fluorescent tails.
